# Peripheral Blur Perception in Young Children at Low Risk or High Risk of Myopia: Longitudinal Data

**DOI:** 10.1167/iovs.66.5.40

**Published:** 2025-05-28

**Authors:** Deepa Dhungel, Peter J. Bex, Aidan McCullough, Iván Marín-Franch, Fuensanta A. Vera-Diaz

**Affiliations:** 1 New England College of Optometry, Boston, Massachusetts, United States; 2 North Eastern University, Boston, Massachusetts, United States; 3 Computational Optometry, Atarfe, Spain; 4 Envision Health Technologies, Inc. Brooklyn, New York, United States; 5 Ophthalmology.World, Madrid, Spain

**Keywords:** peripheral blur perception, blur sensitivity, children's vision, blur discrimination, myopia risk

## Abstract

**Purpose:**

Peripheral sensitivity to blur may contribute to refractive error development. We assessed blur discrimination in the near periphery in emmetropic children (six to eight years) at low risk (LR, *n* = 48) or high risk (HR, *n* =43) for myopia over three years.

**Methods:**

A synthetic image with naturalistic image statistics was digitally blurred (pedestal blur) with either simulated defocus or primary spherical aberration (SA). One quadrant of the display was blurred by an additional amount (increment blur) and the subjects indicated that quadrant. Blur levels were adjusted using QUEST+ adaptive algorithm across three eccentricities (0°, 6°, 12°). Dipper functions were fit to proportions correct as a function of pedestal and increment blur to estimate intrinsic blur and blur criteria. Generalized mixed models were used to describe the effect of eccentricity, visit, and risk group on each blur measure.

**Results:**

There was no significant main effect of risk group, but intrinsic blur decreased significantly more over time in the HR group [defocus: −0.02 log (µm), *P* = 0.015; SA −0.03 log (µm), *P* = 0.005]. Intrinsic blur for defocus [0.11 log (µm), *P* < 0.001], but not SA, was higher for 6° than 0°. Intrinsic blur [defocus: 0.42 log (µm), *P* < 0.001; SA: 0.41 log (µm), *P* < 0.001], and blur criteria (defocus: 0.12 log, *P* < 0.001; SA 0.05 log, *P* = 0.001) were higher for 12° than 0°.

**Conclusions:**

Peripheral blur perception is impaired by elevated intrinsic blur and higher discrimination criteria compared to central vision in children at LR and HR for myopia, with larger transition between 6° and 12° eccentricity. Peripheral blur perception does not appear to be a predictive factor in myopia risk in this small group of children.

Blur is a ubiquitous visual phenomenon that may be defined by different criteria. One common definition of blur is the smearing of an image that leads to a loss of sharpness or clarity.^1^^,^^2^ Blur serves as a cue for driving accommodation and vergence responses, helping to adjust the focus of the eyes and thus obtain clear vision at different distances.^3^^–^^11^ Although some studies have shown conflicting evidence,^12^^,^^13^ along with other near cues, blur also serves as a cue for depth,^13^^–^^17^ with closer targets appearing blurrier than distant targets.^2^ Although the precise mechanisms of emmetropization are not yet understood, blur has been suggested as a causative factor in refractive error development.^18^^–^^30^ The perception of blur is an essential quality of the visual system that allows the processing of varying levels of image sharpness. It is a complex mechanism that involves an interplay of optical, retinal, and neural processing.^6^^,^^15^^,^^31^^–^^33^

The two key components of blur perception include blur detection and blur discrimination.^6^^,^^34^ Blur detection is the minimum amount of blur required for an observer to perceive the presence of image blur relative to a sharp image. In other words, blur detection represents the just noticeable blur. On the other hand, blur discrimination represents the just noticeable difference between different levels of blur.^6^^,^^11^ It is the minimum blur required for an observer to perceive the difference in the blur levels in an image or between two blurry images. Blur discrimination is known to follow a dipper shape^1^^,^^15^^,^^35^^–^^37^ that is characterized by an initial decrease in the discrimination threshold as the baseline blur increases from zero. After the initial decrease, the discrimination threshold increases as the baseline blur continues to increase, resulting in a curve that follows a typical dipper^38^ shape. The point of inflection beyond which the blur discrimination is constrained by extrinsic blur represents the visual system's equivalent intrinsic blur, incorporating both optical and neural blur constraints. Beyond the dip, the dipper curve follows Weber's law.^39^^,^^40^ Watson and Ahumada^1^ provided a detailed review of blur discrimination.

Numerous studies have studied blur perception in the central visual field.^21^^,^^23^^,^^27^^,^^33^^,^^35^^,^^41^^–^^46^ On the other hand, the number of studies that have studied peripheral blur perception is relatively limited,^11^^,^^31^^,^^32^^,^^34^^,^^47^ and, to the best of our knowledge, there have been no studies conducted to assess peripheral blur perception in young children. Studying blur perception in children is particularly important because it has been shown that peripheral blur serves as an optical cue in the process of emmetropization by providing information about the peripheral defocus that can influence axial elongation.^48^^–^^52^ Understanding peripheral blur perception before children develop myopia may also provide insights into the early mechanisms underlying myopia onset.

It has been previously shown that myopic adults may exhibit differences in blur perception compared to emmetropes.^11^^,^^20^^,^^21^^,^^23^^,^^26^^,^^27^^,^^30^ Additionally, it has been reported that optical and neural anisotropy that might affect peripheral blur perception^53^ varies across refractive groups.^54^ Our laboratory has previously studied peripheral blur perception in adults and shown that blur discrimination sensitivity in myopes is lower than in emmetropes, and sensitivity in the visual periphery is lower than in the center.^11^ In this psychophysical experiment, we assessed monocular blur increment discrimination in the near periphery of young children at low or high risk of developing myopia. This experiment is a part of the longitudinal study PICNIC (Preventing myopia: Investigating Contributing factors to Nearsightedness In Children), designed to investigate the factors that influence the development of myopia in young children.^55^

## Methods

### Subjects

One hundred two children between the ages of six and nine years were enrolled in the PICNIC study. Children had functional emmetropia (i.e., emmetropia or low hyperopia at baseline), meaning they did not need to wear optical correction and were tested every six months (±2 weeks), for a total of seven visits over a period of 36 months. Here, we report data from only those children (*n* = 91) who have complete experimental data for a minimum of three visits. Inclusion criteria were uncorrected distance visual acuity 0.10 LogMAR or better in each eye, cycloplegic spherical equivalent (SE) between 0.00D and 2.25D in each eye, with less than 0.75D of astigmatism and less than 0.75D of anisometropia in each eye, and good ocular and general health. Children were classified at baseline as either being at low risk (LR, *n* = 48) or high risk (HR, *n* = 43) for myopia, based on their cycloplegic refractive error and parental myopia.^55^ Throughout the study, children were classified into a Myopia group when their cycloplegic open-field autorefraction spherical equivalent (M) in the right eye became greater than or equal to −0.25D.

The study adhered to the tenets of the Declaration of Helsinki and was approved by the Institutional Review Board at the New England College of Optometry. Informed consent was obtained through written assent from all the participating children along with written consent from their parent or guardian.

### Stimuli and Experimental Apparatus

**Figure 1.**
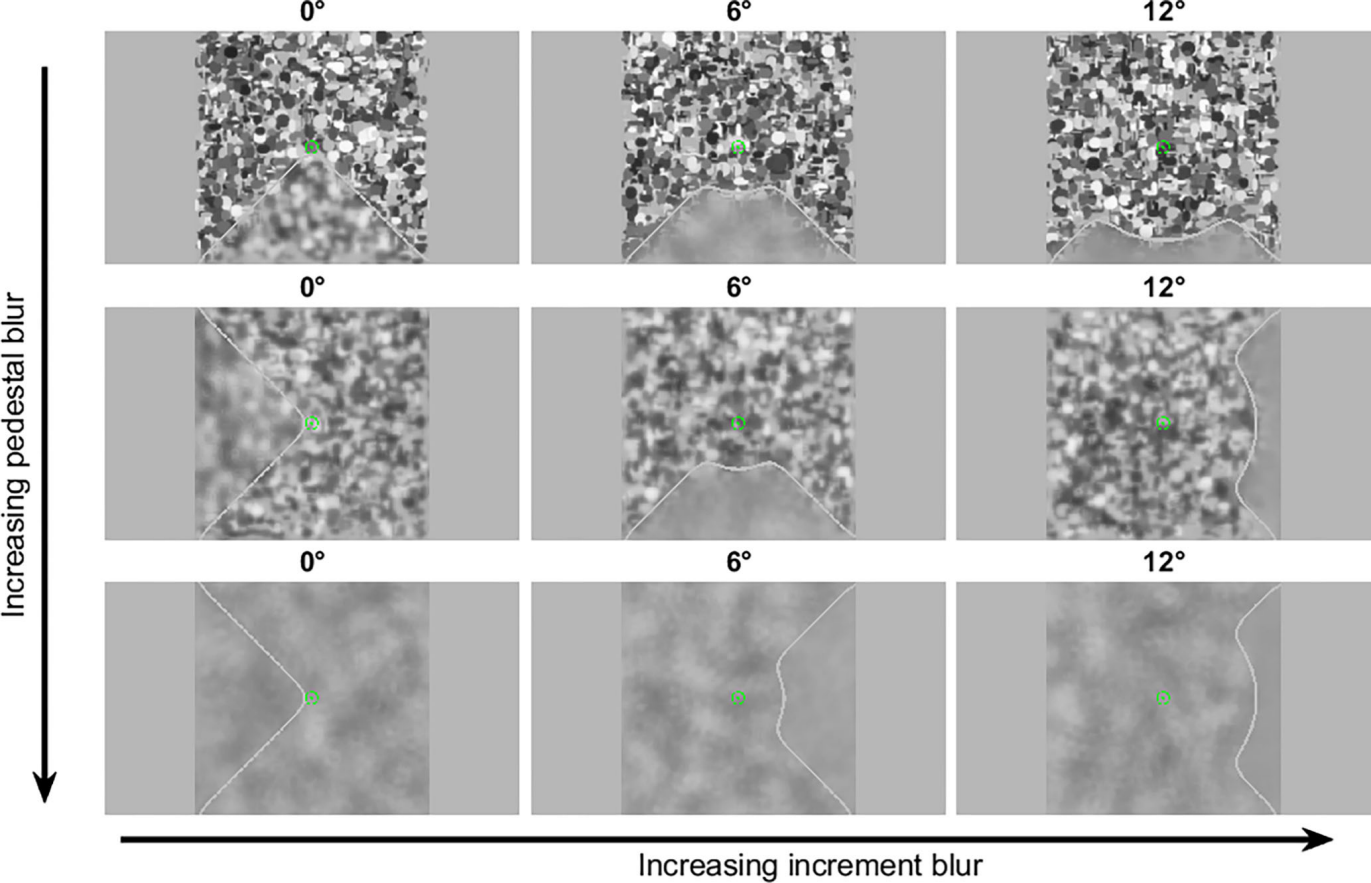
Examples of the dead leaves pattern stimulus for different pedestal blur levels (increasing from top to bottom columns) and increment blur levels (increasing from left to right rows). In this example, the stimuli were digitally blurred with primary SA. A pedestal level of blur was applied to the entire stimulus, and an additional increment blur was applied to an outer target sector. The inner eccentricity of the target sector was either 0°, 6°, or 12° across different, randomly interleaved conditions, the outer eccentricity was the edge of the display. The outlines of the inner sectors in the images shown are marked as *gray* *lines* for illustration purposes only; these lines were not present in the experiment. The two concentric circles that served as the fixation target shown in *green* represent the area in the center of the stimuli that remained sharp. Participants reported with the mouse which of four sectors was blurriest. The fixation targets turned *red* when the subjects made an incorrect response.

Stimuli were 41° × 41° dead leaves patterns (Lee et al., 2001; Bordenave et al., 2006) presented on a gamma-corrected ROG SWIFT PG278Q Asus monitor (Asus Tek Computer Inc., https://www.asus.com/us/) with a resolution of 2560 × 1440 pixels (display dot pitch: 0.233 mm) and a refresh rate of 120 Hz. The monitor subtended a visual angle of 73° × 46° at the viewing distance of 40cm. Examples of the dead leaves pattern stimuli are shown in Figure 1. The dead leaves pattern comprised 2000 ellipses, each with center positions, orientations, aspect ratios, and luminance values drawn from pseudo-random uniform distributions. The semi-axis lengths of the ellipses were randomly selected to fall within a range of 0.1° to 3° of visual angle. The ellipses were layered onto a uniform gray background, allowing overlaps. The dead leaves pattern simulates naturalistic visual environments.^56^

Each image was divided into four 90° sectors: top, right, bottom, and left. The target blur was simulated by transforming the point spread function (PSF) of the image into a frequency domain using the Zernike coefficient using fast Fourier transform (FFT), multiplying it with the image in the frequency domain, and applying an inverse FFT to produce the blurred image reflecting the aberrations defocus [C (2,0)] or primary spherical aberration [C (4,0), i.e., SA]. We specifically chose defocus and primary SA blur because they are known to be different in myopic and emmetropic individuals, and they are thought to play a role in emmetropization.^57^^–^^61^ Numerous studies have reported that myopic individuals have less positive, or more negative, SA.^57^^,^^58^^,^^62^^–^^67^ The PSF of defocus shows a reduced intensity with a softer spread of the peak^68^^–^^71^ whereas the PSF of primary SA shows a reduced intensity of the peak with a broader spread and an increased intensity in the surrounding rings^68^^–^^72^ (Supplementary Fig. S1). Unlike other higher order aberrations, the PSF of SA shows radial symmetry,^68^^,^^70^^,^^71^ affecting the overall sharpness and contrast of the image.

The whole stimulus was blurred by a computer-controlled amount of ‘‘pedestal’’ blur. One of four sectors was blurred by an additional amount of ‘‘increment” blur that extended from 0°, 6°, or 12° eccentricity to the edge of the stimulus across different conditions. Therefore the amount of blur in the test sector was equal to “pedestal + increment” blur. The stimulus included a sharp circular area in the center and two concentric circles of 0.3° and 3° in size, which served as the central fixation target and provided feedback (green for correct, red for incorrect responses). A Tobii EyeX (2017) eye tracker (www.tobii.com) was used to ensure fixation compliance, and trials began only once the participant's gaze was within 2° of the fixation target. Stimuli were presented for 250 msec, which, along with compliant fixation at the start of each trial, ensured stimuli were presented at the required eccentricity.

We used QUEST+,^73^^,^^74^ an adaptive algorithm that adjusts the amount of pedestal and increment blur by minimizing the expected entropy of the posterior probability density. The three test eccentricities and the two types of blur (defocus or SA) were randomly interleaved. The experiment was run for 75 trials for each of the six conditions (3 eccentricities × 2 blur types) for a total of 450 trials. The experimental paradigm was implemented using MATLAB (2015a; The MathWorks, Inc., Natick, MA, USA) with the Psychophysics Toolbox extensions (Version 3).^75^

### Experimental Procedures

As a part of the PICNIC study, standard clinical assessment including thorough ocular health and binocular vision tests, cycloplegic open field autorefraction (WAM-5500, Grand Seiko, www.grandseiko.com/), ocular biometry (Lenstar LS900, HaagStreit, https://haag-streit.com/) was carried out at each visit.^55^ Apart from the peripheral blur discrimination, other experimental tests included static and dynamic accommodation responses, psychophysical contrast sensitivity, retinal responses to simulated blur with, and wide-field posterior segment ocular coherence tomography measurements.

The blur increment discrimination experiment was performed monocularly with the right eye while the left eye was covered with a fogging lens (neutral density filter mounted in spectacle frames). A chin and a forehead rest were used to ensure the subjects’ heads were stabilized. Before the first visit, the subjects practiced at home with an online version of the experimental program implemented with PsyToolkit^76^^,^^77^ while the experimenter monitored the experiment in real time. The experimental procedure was thoroughly explained to the subjects at each visit. Subjects were reminded to keep their fixation at the central fixation target. The experiment began with the calibration of the *Tobii* eye tracker, in which a custom-built nine-point calibration for the 40 cm viewing distance was performed by the participants. The stimulus was presented for 250 ms. The subject's task was to choose the blurriest section of the image by dragging and clicking the mouse in one of the sections of the image: top, right, left, or bottom. Visual feedback was provided by turning the color of the central fixation targets red for incorrect responses and keeping them green for correct responses. If the subjects failed to fixate on the central fixation target, the experiment was interrupted, and a gaze-contingent 0.25° diameter dot was presented at the estimated gaze direction for each eye to alert the participant and the experimenter that fixation was not compliant. When the subject redirected their fixation to the center, the gaze-contingent dots were extinguished, and the trial proceeded. The subjects were allowed to take as many breaks between trials as they wanted.

### Data Analysis

The psychophysics curve fitting and data filtering were carried out using MATLAB 2023a. Dipper functions were fit to estimate the intrinsic blur and blur discrimination criteria for the two blur types and the three eccentricities. The dipper function is described with the equation

(1) $$\begin{array}{c} \Delta b=\sqrt{\left(1+\psi \right)\left(\sigma_{e}^{2}+\sigma_{i}^{2}\right)}-\sigma_{e} \end{array}$$

where σ_e_ represents extrinsic blur (pedestal), σ_i_ represents intrinsic blur, ψ represents the blur discrimination criteria, and Δb represents the blur increment threshold. The intrinsic variance ($\sigma_{i}^{2}$) is the variance due to the presence of intrinsic blur in the observer's visual system, including optical and neural sources. In other words, intrinsic blur represents the inherent blur present in the observer's visual system even in the absence of external blur. A higher intrinsic blur for a subject indicates that even when the image has a very small amount of blur or it is not blurred, the image will be perceived as less sharp (blurrier) than it would be for a person with a lower level of intrinsic blur. The blur discrimination criteria ψ refers to the proportional change in blur required for reliable discrimination between the pedestal blur ($\sigma_{e}^{2}$) added to the intrinsic noise ($\sigma_{i}^{2}$) and the test blur ($\Delta b^{2\;}+\sigma_{e}^{2}$).^40^ In simple terms, it is the just noticeable difference in the variance in blur required for an observer to discriminate between the pedestal blur and the increment blur. A higher level of blur criteria in an observer indicates reduced sensitivity to the difference in blur than that of an observer with lower blur criteria. We applied a binomial probability distribution function to determine the range of chance-level responses. We tested each condition for a total of 75 trials. So, for a guessing rate of 25% (4AFC), the probability of guessing in 24 or more trials is significantly above chance (*P* < 0.05). Therefore we excluded results for which the number of correct responses was less than 24 to remove data where a participant was responding at random; approximately 27% of the data were excluded using this method.

The statistical analysis was performed using R software. Four generalized linear mixed models (GLMMs) with log link functions were used to describe the effect of age at baseline, eccentricity, risk group, and visit number. These models were applied to two outcome variables, namely intrinsic blur and blur discrimination criterion, for each of the two blur types, defocus and SA.

## Results

Age, refractive error M, and axial length (AXL) of the subjects included in the analysis were not normally distributed. Man-Whitney U tests were conducted to test the differences in these variables between the two groups. There were significant differences in age between the LR and HR groups (all *P* < 0.05). The refractive errors, M, were also significantly different between the LR and HR groups at all visits (all *P* < 0.001), as expected. However, AXL was not significantly different between the two groups (all *P* > 0.05). Table 1 shows the descriptive statistics of the age in years (mean ± SD), M component of the cycloplegic refractive error (median ± IQR), and AXL (median ± IQR) for each visit.

**Table 1.** Descriptive Statistics of the Age, Cycloplegic Refractive Error Spherical Equivalent (M), and Axial Length (AXL) for Each Visit

	Age (Years)		M (D)		AXL (mm)	
Visit	LR	HR + Myopia	LR	HR + Myopia	LR	HR + Myopia
1	8.30 ± 1.23	7.24 ± 1.41	1.08 ± 0.56	0.65 ± 0.63	22.75 ± 0.68	22.93 ± 0.71
2	8.75 ± 1.30	7.51 ± 1.53	1.35 ± 0.57	0.55 ± 0.66	22.78 ± 0.72	22.90 ± 0.88
3	9.20 ± 1.25	8.04 ± 1.50	1.27 ± 0.50	0.62 ± 0.79	22.87 ± 0.73	23.11 ± 0.87
4	9.64 ± 1.28	8.61 ± 1.60	1.15 ± 0.53	0.57 ± 0.82	22.94 ± 0.85	23.08± 0.80
5	10.22 ± 1.22	9.12 ± 1.45	1.22 ± 0.45	0.58 ± 0.93	22.95 ± 0.74	23.22 ± 0.73
6	10.67 ± 1.27	9.69 ± 1.46	1.08 ± 0.45	0.49 ± 1.06	23.05 ± 0.79	23.33 ± 0.69
7	11.19 ± 1.24	10.13 ± 1.52	1.14 ± 0.48	0.36 ± 1.16	23.10 ± 0.77	23.52 ± 0.72

Each visit occurred at six months (±2 weeks) from the previous one, for a total of three years. All subjects with myopia were initially classified as HR and are therefore included in the HR group descriptions.

An overall summary of GLMMs is presented in Table 2. The columns represent the independent variables included in the model, only the variables that showed meaningful differences are included in this summary table. The rows represent the two outcome measures for each of the two blur types tested. The coefficients represent the relationship between the outcome measures and the independent variables with the significant values (*P* < 0.05) shown with bold font. Negative coefficients represent a decrease in the outcome measures with increasing independent variables. Technical details and statistical assessments of the individual models can be found in Supplementary Files S2, S3, S4, S5.

**Table 2.** Overall Summary of the Generalized Linear Mixed Models (GLMM) Results

	Risk Group (LR/HR)	Visit	Risk Group/Visit (Interaction)	Eccentricity 6°	Eccentricity 12°
Intrinsic blur defocus [log(µm)]	0.14 (*P* = 0.062)	**−0.10 (*P*** **= 0.009)**	**−0.02** **(*P*** **= 0.015)**	**0.11 (*P*** **< 0.001)**	**0.42 (*P*** **< 0.001)**
Intrinsic blur SA [log(µm)]	0.14 (*P* = 0.108)	**−0.11 (*P*** **< 0.001)**	**−0.03** **(*P*** **= 0.005)**	0.01 (*P* = 0.639)	**0.41 (*P*** **< 0.001)**
Log blur criteria defocus (log units)	0.03 (*P* = 0.387)	**−0.004 (*P*** **< 0.001)**	**−**0.00 (*P* = 0.601)	0.01 (*P* = 0.181)	**0.12 (*P*** **< 0.001)**
Log blur criteria SA (log units)	0.09 (*P* = 0.216)	**−0.10** **(*P*** **= 0.007)**	**−**0.01 (*P* = 0.199)	0.02 (*P* = 0.236)	**0.05 (*P*** **= 0.007)**

Values marked in bold font indicate significant effects (*P* < 0.05).

Although there was no significant main effect of risk group on any of the outcome measures: (all *P* > 0.05), there was a significant interaction between risk group and time (visit number) for intrinsic blur for both defocus [−0.02 log (µm), *P* = 0.015] and SA [−0.03 log (µm), *P* = 0.005]. This suggests that intrinsic blur for both defocus and SA in the children in the HR group decreased slightly more over time compared to those in the LR group. The interaction effect for blur discrimination criteria did not reach significance for both defocus and SA.

**Figure 2.**
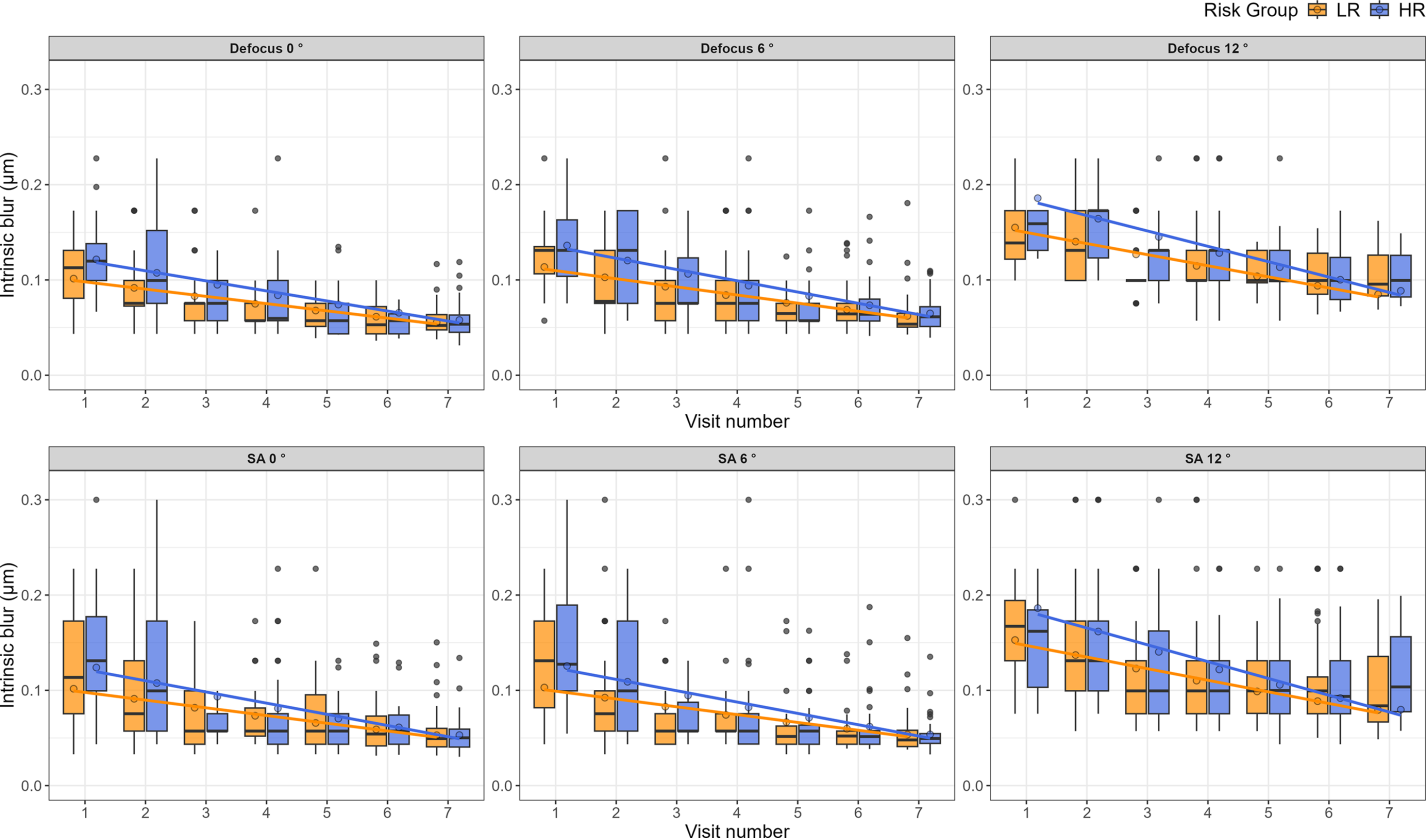
Boxplots showing intrinsic blur for defocus (*top row*) and SA (*bottom row*) across visits for LR (*orange boxes*) and HR (*blue boxes*) groups. The lines color-coded for risk group represent the linear best fit to the estimates from the GLMM. The *black midlines* show the median, *boxes* show the interquartile range, *whiskers* show 95% fiducial limits, and *data points* show outliers. For both defocus and SA, intrinsic blur and the difference in intrinsic blur between the LR and HR decrease over time for all eccentricities.

There was a significant decrease in the intrinsic blur with age at baseline for both defocus [−0.10 log (µm), *P* = 0.009] and SA [−0.14 log (µm), *P* = 0.001] but not for blur discrimination criteria, indicating a lower intrinsic blur for older subjects (Supplementary Tables S1, S2, S3 and S4). There was a significant decrease in intrinsic blur with time, over subsequent visits for both defocus [−0.10 log (µm), *P* < 0.001] and SA [−0.11 log (µm), *P* < 0.001]. Additionally, blur discrimination criteria showed a decrease for both defocus (−0.04 log units, *P* < 0.001) and SA (−0.10 log units, *P* < 0.001). These improvements (a decrease in intrinsic blur and a decrease in the just noticeable difference required for discrimination) indicate a potential learning or developmental effect. Figure 2 shows the change in intrinsic blur with time for defocus in the top row and SA in the bottom row. Each panel represents a different eccentricity, increasing from left to right across the columns. The lines, color-coded for the corresponding risk groups, represent the linear fit to the GLMM estimates.

**Figure 3.**
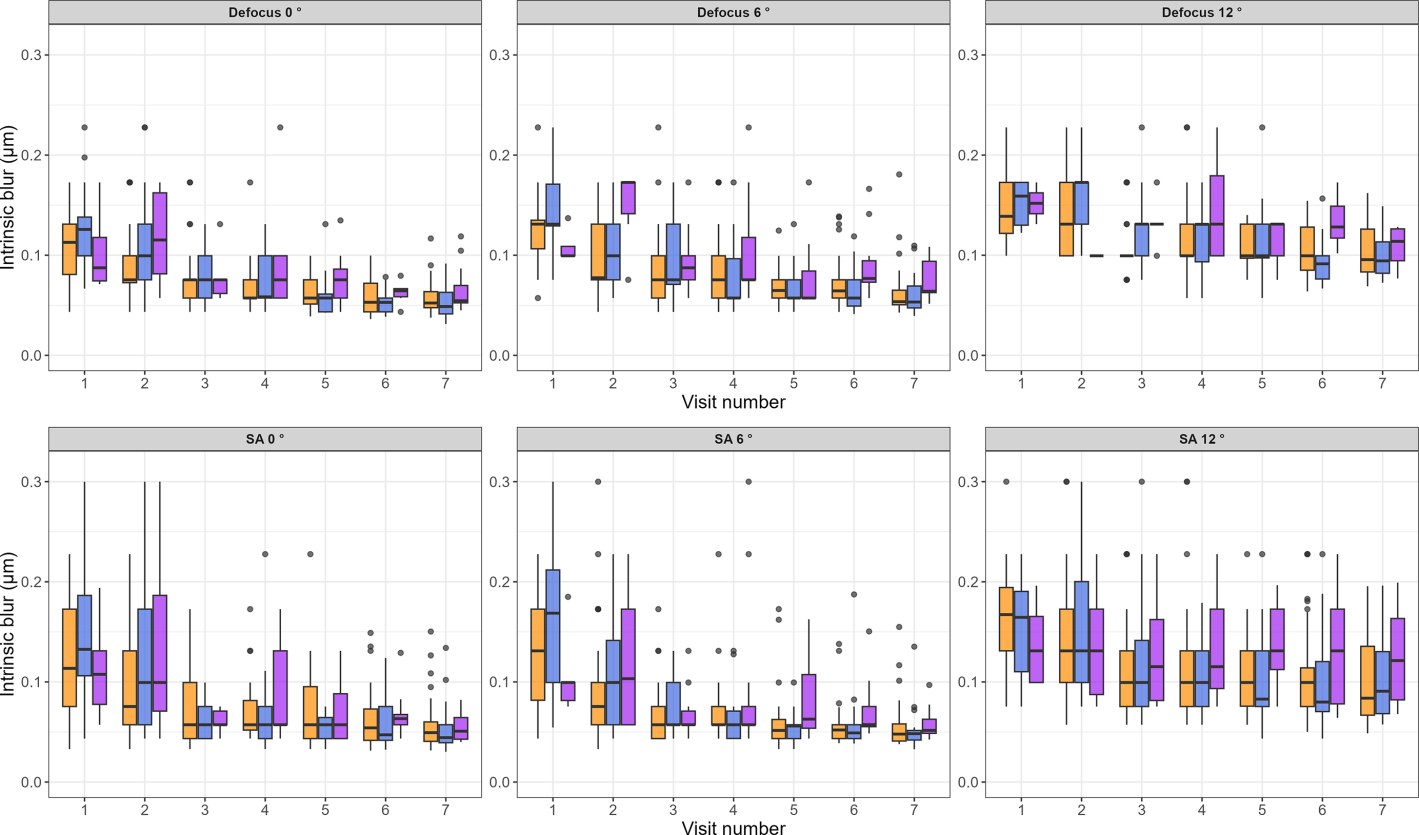
Boxplots showing intrinsic blur for defocus (*top row*) and SA (*bottom row*) across visits for LR (*orange boxes*) group, the HR group—without the subjects who developed Myopia (*blue boxes*), and the Myopia (*purple* *boxes*) group. The Myopia group appears to show overall lower intrinsic blur levels than the LR and HR group at baseline (visit 1), which seems to disappear with time. The changes with time in the Myopia group did not show a clear trend, possibly because of a small number of subjects.

**Figure 4.**
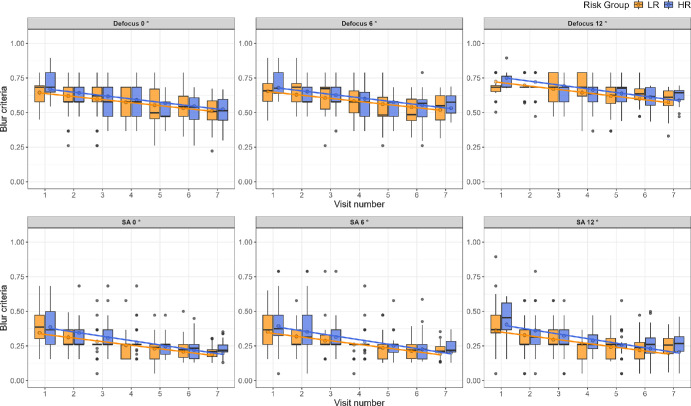
Boxplots showing blur discrimination criteria for defocus (*top row*) and SA (*bottom row*) for LR (*orange boxes*) and HR (*blue boxes*) groups with time as in Figure 2, except for blur discrimination criteria. For both defocus and SA, blur criterion and the difference in blur criterion between the LR and HR decreases over time for all eccentricities.

Figure 3 shows the change in intrinsic blur with time for defocus, with the Myopia subgroup plotted separately, shown in purple. Although the Myopia subgroup appears to show overall lower intrinsic blur levels than the LR and HR groups at baseline (visit 1) for both Defocus and SA—an effect that seems to disappear with time, these trends are not strong enough to draw definitive conclusions. The small and varying number of subjects in the different test conditions provides insufficient information. A total of 14 subjects in the HR group and none in the LR group developed myopia during the course of the study. However, the number of subjects with reliable data in each test condition varied, making reliable statistical testing with Myopia as a subgroup not feasible. For these reasons, we performed the statistical modeling between the HR and LR groups without dividing the HR into the HR only and the myopia subgroup. Figure 4 shows the change in blur discrimination criteria with time for defocus in the top row and SA in the bottom row, following the same color scheme as Figure 2.

**Figure 5.**
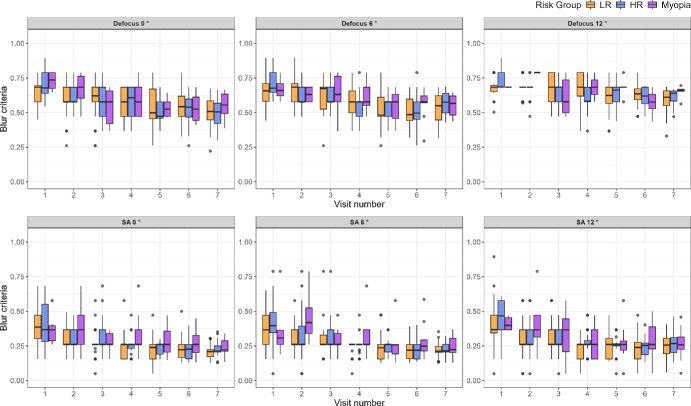
Boxplots showing blur criteria for defocus (*top row*) and SA (*bottom row*) across visits for the LR (*orange boxes*) group, the HR group—without the subjects who developed Myopia (*blue boxes*), and the Myopia (*purple* *boxes*) group. There were no clear trends showing differences between the Myopia and LR or HR groups, although there appears to be higher levels of blur criteria for defocus 0° at some visits, not for defocus 6° or 12°. Blur criteria decreased over time for all eccentricities, with no clear differences between the Myopia and LR or HR groups.

The increase in intrinsic blur at 6° eccentricity compared to 0° reached significance for defocus only [0.11 log (µm), *P* < 0.001]. However, when comparing 0° to 12° eccentricity, all the outcome measures: intrinsic blur for defocus [0.42 log (µm), *P* < 0.001], intrinsic blur for SA [0.41 log (µm), *P* < 0.001], blur discrimination criterion for defocus (0.12 log units, *P* < 0.001) and blur discrimination criterion for SA (0.05 log units, *P* = 0.007) were significantly higher. Pairwise comparison with Bonferroni adjustment revealed that intrinsic blur for defocus at 12° was significantly higher than at 6° eccentricity [−0.31 log (µm), *P* < 0.001]. Intrinsic blur for SA at 12° was also higher compared to 6° eccentricity [−0.39 log (µm), *P* < 0.001]. Blur discrimination criterion for defocus (−0.1 log units, *P* < 0.001) at 12° was significantly higher than 6° eccentricity, this effect was not seen for SA. The effect of eccentricity on the outcome measures for each visit are illustrated in Supplementary Figures S2, S4, S6, and S8. Blur criteria decreased over time for all eccentricities, and there were no clear differences between the Myopia and LR or HR groups, with some possible trends for Defocus, as shown in Figure 5. The limited number of participants in the Myopia subgroup did not permit statistical analysis.

Pearson correlation analysis revealed no significant correlation between AXL to anterior corneal radius (AXL/CR) and intrinsic blur and blur discrimination criteria in most cases. There was weak but significant negative correlation between intrinsic blur and AXL/CR for blur extending beyond 12° at visit 3 (*r* = −0.343, *P* = 0.015) and weak but significant positive correlation at visit 6 (*r* = 0.287, *I* = 0.041). Intrinsic blur for SA showed significant correlation with AXL/CR for blur extending up to the fovea for visit 4 (*r* =0.308, *P* = 0.007) and visit 7 (*r* =0.232, *P* = 0.046). There was a weak but statistically significant positive correlation between AXL/CR with blur criterion for defocus for eccentricity beyond 12° for visit 2 (*r* = 0.382, *P* = 0.009) and visit 5 (*r* = 0.391, *P* = 0.003) and with blur criteria for SA for eccentricity beyond 12° (*r* = 0.254, *P* = 0.030) at visit 3. Supplementary Figures S10 through S13 show the correlation analysis.

Similarly, the correlation between refractive error, M, and intrinsic blur and blur discrimination criteria did not show a significant correlation in most cases. There was only a marginal significance in correlation of M with intrinsic blur for defocus at visit 6 for eccentricity beyond 12° (*r* = −0.276, *P* = 0.047) and weak but statistically significant positive correlation with blur criteria for SA for blur extending up to fovea (*r* = 0.26, *P* = 0.039) at visit 7. Supplementary Figures S14 through S17 show the correlation analysis.

## Discussion

In a previous study, we compared peripheral blur perception in young adults with emmetropia or myopia using a similar paradigm to that used in the present study, and we found an increase in intrinsic blur and a decrease in blur sensitivity in the periphery compared to the center. Additionally, the monocular blur sensitivity in myopes was significantly worse than in the emmetropes.^11^ We were able to successfully adapt the experimental design to test young children in this study. A few modifications were made to the experimental paradigm. Whereas the previous study used digital Gaussian blur, the current approach used digital filtering that simulated defocus and SA to mimic the blur created by these aberration terms that are likely relevant to developmental eye growth and myopia.^57^^–^^61^ It is worth noting that targets in this study were blurred using Zernike coefficients for a 5 mm pupil. These may be converted to Seidel defocus in diopters (D) and Seidel SA (in D per mm square from the pupil center, D/mm^2^) for a more clinical interpretation by a simple linear transformation.^78^ Specifically, since the pupil is 5 mm in diameter, then Seidel defocus is -1.1 times Zernike defocus [C(2,0)] in D and Seidel SA is 1.4 times Zernike primary SA [C(4,0)] in D/mm^2^. This transformation, however, did not affect the key results and interpretation of the models. We tested 0°, 6°, and 12° eccentricities instead of the 0°, 4°, 8°, and 12° eccentricities used in the earlier study because we previously observed differences in blur processing beyond 4° only. We limited our testing to the near because the active site for refractive development in primate retina is shown to be within 20°, with imposed optical defocus showing more prominent effect within 15° eccentricity.^51^ We tested only three eccentricities to shorten the experimental duration for the children. In this longitudinal study with young children, we were able to assess blur discrimination, providing insights into how blur perception may change during development.

The results of the current study confirm that blur processing deteriorates in the near periphery in children, similar to what was observed in young adults.^11^ Using an additive noise analysis, we can isolate differences in blur processing that are due to internal noise and those that are due to criterion changes or undersampling.^79^^,^^80^ The results show that intrinsic blur for defocus was significantly higher for 6° compared to 0° and increased further at 12° eccentricity. Although intrinsic blur for SA was not significantly different at 6° compared to 0°, it was significantly higher at 12° compared to 6°. The higher intrinsic blur levels found for eccentricities further away from the fovea indicate that the internal noise in the system is higher further in the periphery, and thus a higher level of external blur is required for the observer to perceive the external blur. Likewise, blur discrimination criterion for defocus was also higher at 12° compared to 6° eccentricity, indicating that further in the periphery, the observer's ability to discriminate blur (additional blur in the presence of background blur) is reduced, and the observer has reduced sensitivity to the difference in blur. All the outcome measures were significantly higher for 12° eccentricity compared to 0° (blur reaches the fovea). Since all the outcome measures except the blur discrimination criterion for SA were significantly higher for 12° eccentricity than 6°, the visual area between 6° and 12° seems to show the largest effect in blur processing. This outcome aligns with the findings from a previous electrophysiological study on blur processing in adults using similar stimuli by our group.^81^

Our findings clearly indicate that there is an effect of eccentricity on blur processing in children. Such an effect of eccentricity on blur perception has been reported by previous studies^11^^,^^31^^,^^32^^,^^34^^,^^47^ and can be attributed to various factors, including the substantial decrease in photoreceptors and retinal ganglion cell density outside of the fovea^31^^,^^32^^,^^34^^,^^47^^,^^82^^,^^83^ and the neurophysiology of the visual cortex.^31^^,^^32^^,^^46^^,^^84^^,^^85^ Besides, the optics of the eye also become worse in the periphery compared to the center.^86^^,^^87^ Wang and Ciuffreda^31^ have pointed out visual attention^88^^,^^89^ and sharpness overconstancy^90^ as potential contributing factors to deterioration in peripheral blur perception as well. Sharpness overconstancy is a perceptual phenomenon because of which blurred images in the periphery appear sharper than they actually are.^90^ The design of the present stimulus was such that the central 6° and 12° portion of the stimulus was blurred at the pedestal level, and only the sector beyond the target eccentricity contained additional blur. Because the central portion of the stimulus was blurred at the pedestal level, and because the visual system prioritizes the visual information from the center over the periphery,^91^ sharpness overconstancy might provide a descriptive explanation (but not a mechanism) for the decrease in blur perception in the periphery in this study. Furthermore, we noted that the rate of increase in intrinsic blur and the blur discrimination criterion from 6° to 12° eccentricity was higher than from 0° to 6° eccentricity. This finding indicates that, similar to other visual functions,^92^^–^^95^ the relationship between blur perception and visual eccentricity is not linear, just as it is not a linear function of pedestal blur. Peripheral blur perception may likely benefit from accounting for the cortical magnification factor.^95^^–^^98^

Peripheral visual performance has been reported to decrease more sharply in adults with myopia compared to those with emmetropia.^99^^,^^100^ Moreover, because adult myopes in our previous experiment exhibited poorer blur perception than the emmetropes,^11^ our initial hypothesis was that children who are functionally emmetropic would show a difference in peripheral blur perception depending on their risk for myopia (HR or LR). This hypothesis is supported by the notion that the peripheral hyperopic defocus is a contributing factor to myopia development.^48^^,^^50^^–^^52^ However, our results did not show a significant main effect of the risk group, suggesting that there was no overall difference in blur perception in young children between the two groups. There was a trend for a marginal effect of risk group on intrinsic blur for defocus [0.14 log (µm), *P* = 0.062], showing a higher intrinsic blur for HR than the LR group. However, it could be an effect of more negative spherical equivalent refractive error in the HR group. Studies examining the depth of focus at the fovea in adults have reported conflicting findings. Rosenfield and Abraham-Cohen^21^ reported a significantly weaker sensitivity to optical blur in myopes than emmetropes. Similarly, George and Rosenfield^44^ observed a weaker blur adaptation response in myopes compared to emmetropes at low contrast levels. Wang et al. ^46^ reported that blur sensitivity after adaptation to optical blur is reduced in myopes compared to pre-adaptation. However, their subject pool did not include non-myopes. Cufflin et al.^27^ compared blur sensitivity following adaptation in myopes and emmetropes and found no significant difference between the effect of blur adaptation on blur discrimination thresholds in myopes compared to emmetropes. Mankowska et al.^101^ extended this lack of difference in blur sensitivity between emmetropes and myopes in the parafovea.

There have been only a limited number of studies on blur perception in children, and even fewer on the periphery. To the best of our knowledge, there is no previous study on peripheral blur discrimination in children. Schmid et al.^23^ reported similar foveal blur detection thresholds for myopic and nonmyopic children when tested with various naturalistic images blurred across a range of digital defocus blur. Labhishetty et al.^102^ confirmed the findings using white-on-black lines as stimuli for varying levels of defocus blur. From our study we report that blur discrimination was not different between the children at low risk and high risk of myopia. When we added the interaction between eccentricity and risk group in the GLMMs, we did not find a significant interaction between them, indicating that the blur processing in the two groups was similar not just in the center but also in the near periphery. Together, these findings suggest that blur perception might not be a predictive factor in myopia risk.

Nonetheless, there were significant interaction effects of risk group and visit for intrinsic blur for both defocus and SA. Specifically, the rate of decrease in intrinsic blur for the HR group was higher than the rate of decrease in intrinsic blur in the HR group. This indicates that the development of blur processing might vary in the HR compared to the LR group. Although the age at baseline was taken into account, it is also worth noting that there was a significant difference in the refractive error at baseline between the two groups, which might affect the development of blur processing. There was a significant decrease in intrinsic blur (for both defocus and SA) with time, suggesting that blur perception may improve with age or practice.

This study has some limitations. First, a significant portion of the data had to be excluded because the data were not reliable. The complex study design with a total of 12 combinations: six outcome measures (three eccentricities and two blur types) for two risk groups across seven visits complicated the statistical model fitting. Similarly, a small number of subjects in each group posed another limitation. The use of Zernike primary SA, [C (4,0)] means that the SA-blurred targets include not only Seidel SA but also Seidel defocus. The amount of defocus added is, in fact, 4.3 times SA [C (4,0)] in D. On average, the simulation and thresholds had 0.18 D/mm^2^ of Seidel SA and 0.55D of Seidel defocus. Future studies relevant to refractive error development should consider using Seidel coefficients for simulated blur. Nevertheless, we were able to replicate the main findings from the adults pertaining to the effect of eccentricity on blur perception and extend the findings to defocus and spherical aberrations. Future studies should consider comparing perceptual blur discrimination in myopic and emmetropic children.

In conclusion, peripheral blur perception is impaired by elevated levels of intrinsic blur and higher discrimination criteria than central vision in children at LR and HR of myopia. However, peripheral blur perception does not appear to be a predictive factor in myopia risk. The results suggest that blur perception may develop differently in low and high risk groups, with the HR group showing a decrease in intrinsic blur at a higher rate than the LR group.

## Supplementary Material

Supplement 1

Supplement 2

Supplement 3

Supplement 4

Supplement 5

Supplement 6

Supplement 7
