# Supplementary material for: Peripheral Blur Perception in Young Children at Low Risk or High Risk of Myopia: Longitudinal Data
Source: Invest Ophthalmol Vis Sci. 2025 May 28;66(5):40. doi: 10.1167/iovs.66.5.40 (PMC12126130; doi:10.1167/iovs.66.5.40)
Supplement: Supplement 1 [file iovs-66-5-40_s001.pdf]

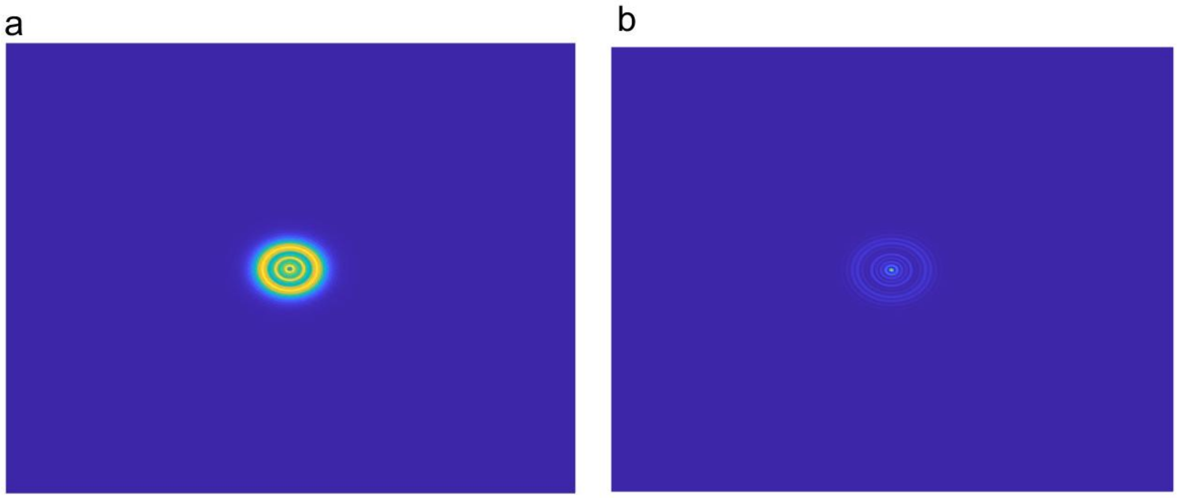

Supplementary Figure S1: Examples of PSF of Defocus (a) and SA(b) of the same amount in the absence of other higher order aberrations. The point spread function (PSF) of defocus shows a reduced intensity with a softer spread of the peak, whereas primary SA shows a reduced intensity of the peak with a broader spread of the peak and an increased intensity in the surrounding rings.
