# Supplementary material for: Peripheral Blur Perception in Young Children at Low Risk or High Risk of Myopia: Longitudinal Data
Source: Invest Ophthalmol Vis Sci. 2025 May 28;66(5):40. doi: 10.1167/iovs.66.5.40 (PMC12126130; doi:10.1167/iovs.66.5.40)
Supplement: Supplement 2 [file iovs-66-5-40_s002.pdf]

Intrinsic blur for defocus

The detailed model summary is shown in Supplemental Table S1. The values are in log(µm) units. Significant effects(p<0.05) are highlighted in bold.

Supplementary Table S1:

Summary of the GLMM fitted to the intrinsic blur for defocus.

	Intrinsic blur for defocus		
Predictors	*Estimates*	*CI*	*P*
(Intercept)	-1.44	-2.01 – -0.87	**<0.001**
Age at baseline	-0.10	-0.17 – -0.02	**0.009**
Risk group [1]	0.14	-0.01 – 0.29	0.062
Eccentricity [6]	0.11	0.07 – 0.15	**<0.001**
Eccentricity [12]	0.42	0.39 – 0.46	**<0.001**
Visit	-0.10	-0.11 – -0.09	**<0.001**
Risk group: Visit	-0.02	-0.04 – -0.00	**0.015**
**Random Effects**			
σ^2	0.00		
$\tau_{00\ Subject}$	0.02		
ICC	0.96		
$N_{Subject}$	97		
Observations	1203		
Marginal R^2 / Conditional R^2	0.791 / 0.993		

There was a significant increase in intrinsic blur in intrinsic blur at 6° eccentricity [0.11 log (µm), p < 0.001] and a greater increase at 12° eccentricity [0.42 log (µm), p < 0.001] compared to fovea. Pairwise comparison with Bonferroni adjustment revealed that intrinsic blur for defocus

at 12° was significantly higher than at 6° eccentricity [-0.31log (μm), p<0.001]. There was a significant decrease in the intrinsic blur with age at baseline (-0.10 log (μm), p = 0.009) indicating a lower intrinsic blur for older subjects. There was also a significant decrease in intrinsic blur with time, over subsequent visits [-0.10 log (μm), p < 0.001], indicating a potential learning or developmental effect. While the main effect of risk group did not reach significance [0.14 log (μm), p = 0.062], there was a significant interaction between risk group and time (visit number) [-0.02log (μm), p = 0.015], suggesting that intrinsic blur for defocus for the children at HR of myopia decreased slightly more over time compared to the LR.

Supplemental Figure S2 shows boxplots for intrinsic blur for defocus plotted across eccentricities (degrees) with orange boxes representing the LR and blue representing the HR group. Each panel represents a different visit, labeled with visit numbers 1 through 7. The number of included subjects for each eccentricity is indicated by the corresponding color-coded numbers on the top of each panel. The lines color-coded for the corresponding risk groups represent the GLMM estimates.

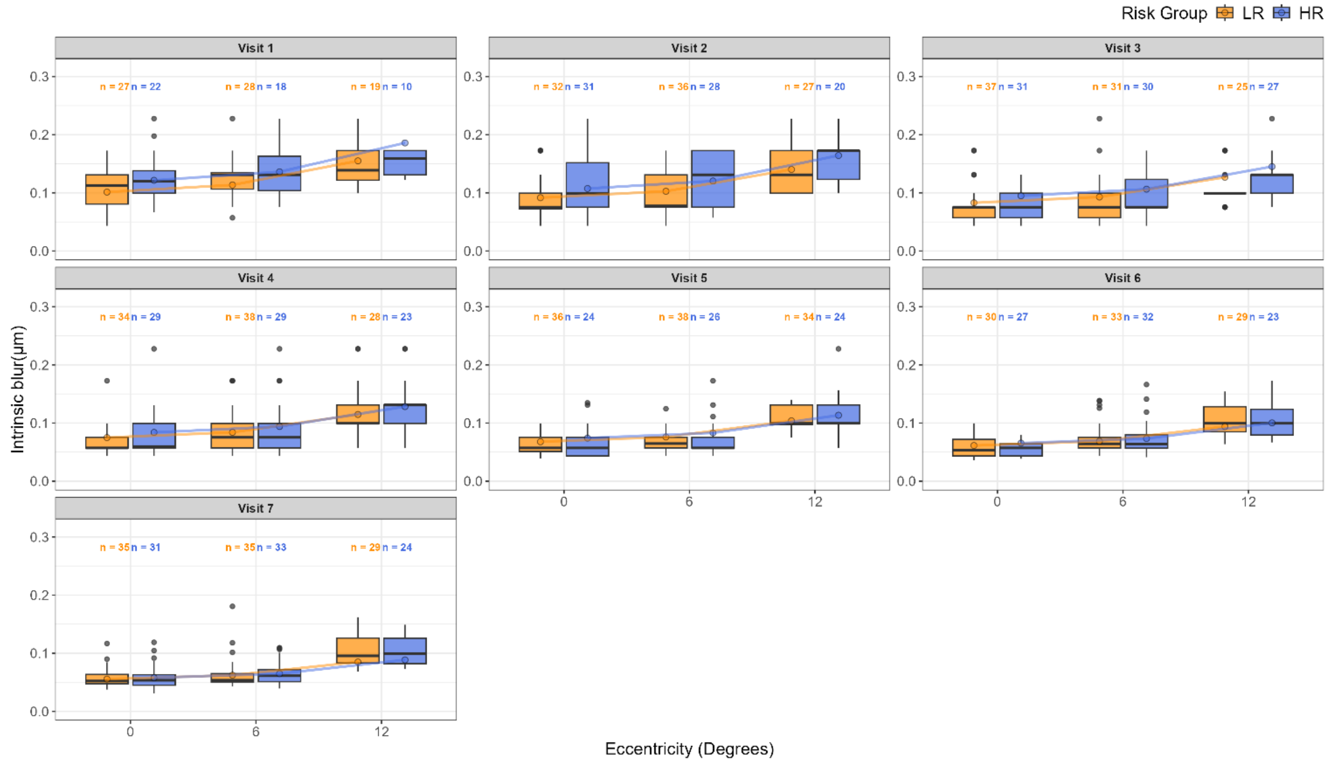

Supplementary Figure S2: Boxplots showing intrinsic blur for LR (orange boxes) and HR (blue boxes) groups for defocus blur. The black midline shows the median, boxes show the interquartile range, whiskers show 95% fiducial limits, and data points show outliers. The lines represent the estimates from the GLMM. At all visits, intrinsic blur was higher for the peripheral targets compared to the fovea.

Supplemental Figure S3 (a) shows Q-Q plot and (b) shows the scatter plots of the Pearson residuals of the models. The points (black circles) on the Q-Q plot deviate significantly from the diagonal reference line (shown in red). We acknowledge that the heavier right tail in the distribution of the residual deviates from the ideal normal distribution. However, after testing several link functions, the current model provided the best fit for the raw data. More importantly, the estimates generated by the model matched the raw values closely as shown by the linear fits to the estimates in Supplemental figure S2.

The residuals [Supplemental Figure S3 (b)] show a periodic pattern and a deviation of the correlation line (blue) from the reference line (red) which might indicate that the model might not fully capture the systemic variations in the data. However, the patterns can be attributed to the decision-making process in the QUEST+ algorithm. Additionally, high marginal R^2 (0.79) and high conditional R^2 (0.99) indicate strong predictability of the model.

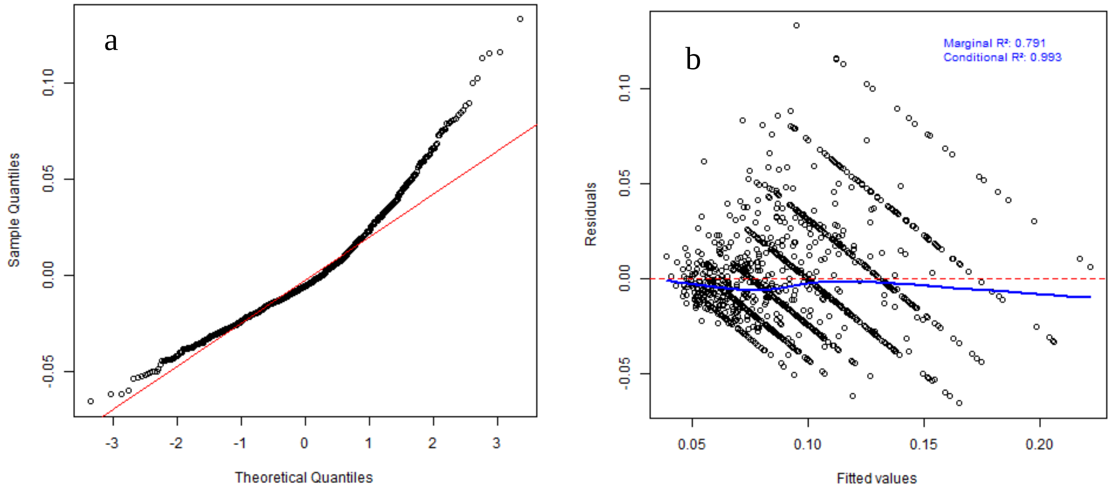

Supplementary Figure S3: Q-Q plot (a) and scatter plot (b) of the Pearson residuals of GLMM fitted intrinsic blur for defocus.
