# Supplementary material for: Peripheral Blur Perception in Young Children at Low Risk or High Risk of Myopia: Longitudinal Data
Source: Invest Ophthalmol Vis Sci. 2025 May 28;66(5):40. doi: 10.1167/iovs.66.5.40 (PMC12126130; doi:10.1167/iovs.66.5.40)
Supplement: Supplement 3 [file iovs-66-5-40_s003.pdf]

Intrinsic blur for SA

Supplemental Table S2 shows the model summary for intrinsic blur for SA. The values are in log(µm) units.

Supplementary Table S2:

Summary of the GLMM fitted to the intrinsic blur for defocus.

	**Intrinsic blur for SA**		
Predictors	*Estimates*	*CI*	*p*
(Intercept)	-1.08	-1.74 – -0.42	**0.001**
Age at Baseline	-0.14	-0.22 – -0.06	**0.001**
Risk group [1]	0.14	-0.03 – 0.31	0.108
Eccentricity [6]	0.01	-0.04 – 0.07	0.639
Eccentricity [12]	0.41	0.36 – 0.45	**<0.001**
Visit	-0.11	-0.12 – -0.09	**<0.001**
Risk group: Visit	-0.03	-0.06 – -0.01	**0.005**
**Random Effects**			
σ^2	0.00		
$\tau_{00\ Subject}$	0.03		
ICC	0.95		
$N_{Subject}$	98		
Observations	1580		
Marginal R^2 / Conditional R^2	0.788 / 0.989		

There was a significant increase in intrinsic blur in intrinsic at 12° eccentricity [0.41 log (µm), p < 0.001] but not at 6° eccentricity [0.01 log (µm), p = 0.639] compared to fovea. Post-hoc pairwise comparison showed a significantly higher intrinsic blur for SA at 12° compared to 6° eccentricity [-0.39 log(µm), p<0.001]. There was a significant decrease in the intrinsic blur with age at baseline [-0.14 log (µm), p = 0.001] and also with time, over subsequent visits [-0.11 log

(μm), p < 0.001]. As in the case of intrinsic blur for defocus, there was a significant interaction between risk group and time (visit number) for SA [-0.03 log (μm), p=0.005] with no main effect of risk group, suggesting that intrinsic blur for SA for children at HR of myopia decreased slightly more over time compared to the LR.

Supplemental Figure S4 shows boxplots for intrinsic blur for SA plotted across eccentricities (degrees) with orange boxes representing the LR and blue representing the HR group. The figure follows the same color scheme as Supplemental Figure S1. The detailed model summary is shown in Supplemental Table S2 and the corresponding residual plot and Q-Q plot in Supplemental Figures S5(a) S5(b).

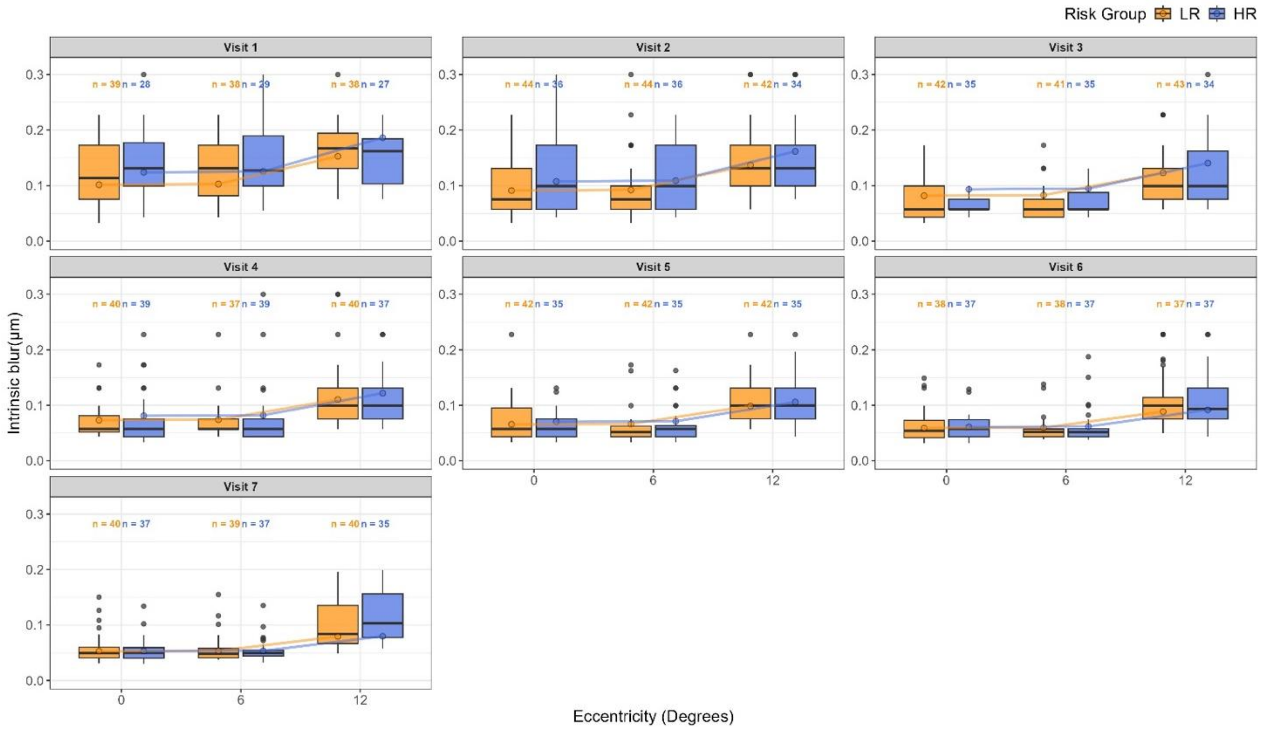

Supplementary Figure S4: Boxplots showing intrinsic blur for LR (orange boxes) and HR (blue boxes) groups for SA blur, as in Supplemental Figure S1, except for SA. The lines represent the estimates from the GLMM. The intrinsic blur was higher for the peripheral targets compared to the fovea.

Supplemental Figure S5 (a) shows Q-Q plot and (b) shows the scatter plots of the Pearson residuals of the models. The points (black circles) on the Q-Q plot deviate significantly from the diagonal reference line (shown in red) showing a heavy right tail. The residuals show a periodic pattern and a deviation of the correlation line (blue) from the reference line(red). See Supplemental File S2 for more details. The estimates generated by the model matched the raw values closely as shown by the linear fits to the estimates in Supplemental Figure S3. Marginal R^2 of 0.79 and conditional R^2 0.99 indicate strong predictability of the model.

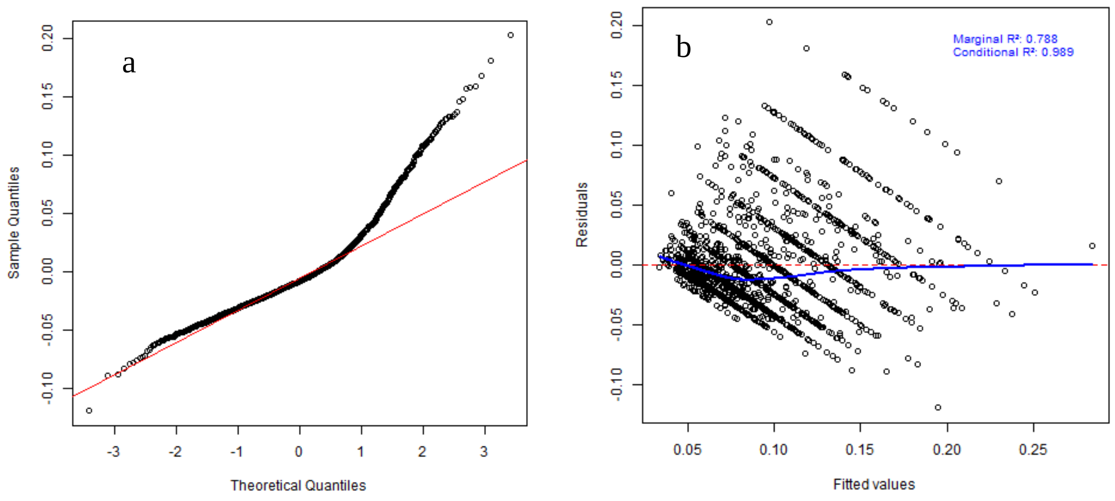

Supplementary Figure S5: Q-Q plot (a) and scatter plot (b) of the Pearson residuals of GLMM fitted intrinsic blur for SA.
