# Supplementary material for: Peripheral Blur Perception in Young Children at Low Risk or High Risk of Myopia: Longitudinal Data
Source: Invest Ophthalmol Vis Sci. 2025 May 28;66(5):40. doi: 10.1167/iovs.66.5.40 (PMC12126130; doi:10.1167/iovs.66.5.40)
Supplement: Supplement 4 [file iovs-66-5-40_s004.pdf]

Blur discrimination criterion for defocus

Supplemental Table S3 shows the model for blur discrimination criteria for defocus. The values are in log units. It is worth noting that the model for blur discrimination criterion for defocus failed to converge, but we have kept the model because it showed reasonable estimates, as depicted by the solid lines in Supplemental Figure S4.

Supplementary Table S3:

Summary of the GLMM fitted to the blur discrimination criterion for defocus *Model failed to converge

	**Blur discrimination criterion for defocus**		
Predictors	*Estimates*	*CI*	*p*
(Intercept)	-0.27	-0.52 – -0.03	**0.026**
Age at baseline	-0.02	-0.05 – 0.01	0.300
Risk group [1]	0.03	-0.04 – 0.10	0.387
Eccentricity [6]	0.01	-0.01 – 0.04	0.181
Eccentricity [12]	0.12	0.09 – 0.14	**<0.001**
Visit	-0.04	-0.04 – -0.03	**<0.001**
Risk group: Visit	-0.00	-0.01 – 0.01	0.601
**Random Effects**			
σ^2	0.01		
$\tau_{00\ Subject}$	0.00		
ICC	0.32		
$N_{Subject}$	97		
Observations	1203		
Marginal R^2 / Conditional R^2	0.382 / 0.582		

There was a significant increase in blur discrimination criterion for defocus at 12° eccentricity (0.12 log units, $p < 0.001$) but not at 6° eccentricity (0.01 log units, $p = 0.18$) compared to fovea. Blur discrimination criterion for defocus for 12° was significantly higher than 6° eccentricity (-0.1 log units, $p<0.001$) on pairwise comparison. There was a significant decrease in the blur discrimination criteria over time, subsequent visits (-0.04 log units, $p < 0.001$), but not with age at baseline (-0.02 log units, $p =0.3$). There was no significant main effect of risk group (0.03 log units, $p =0.39$) or interaction between risk group and time (visit number) (-0.00 log units, $p =0.60$).

Supplemental Figure S6 shows boxplots, orange for the LR and blue for the HR group with the blur discrimination criterion for defocus plotted across the eccentricity (degrees). The figure follows the same color scheme as Supplemental Figure S2. The lines represent the estimates from the GLMM. The blur criterion was higher for the peripheral targets compared to the fovea.

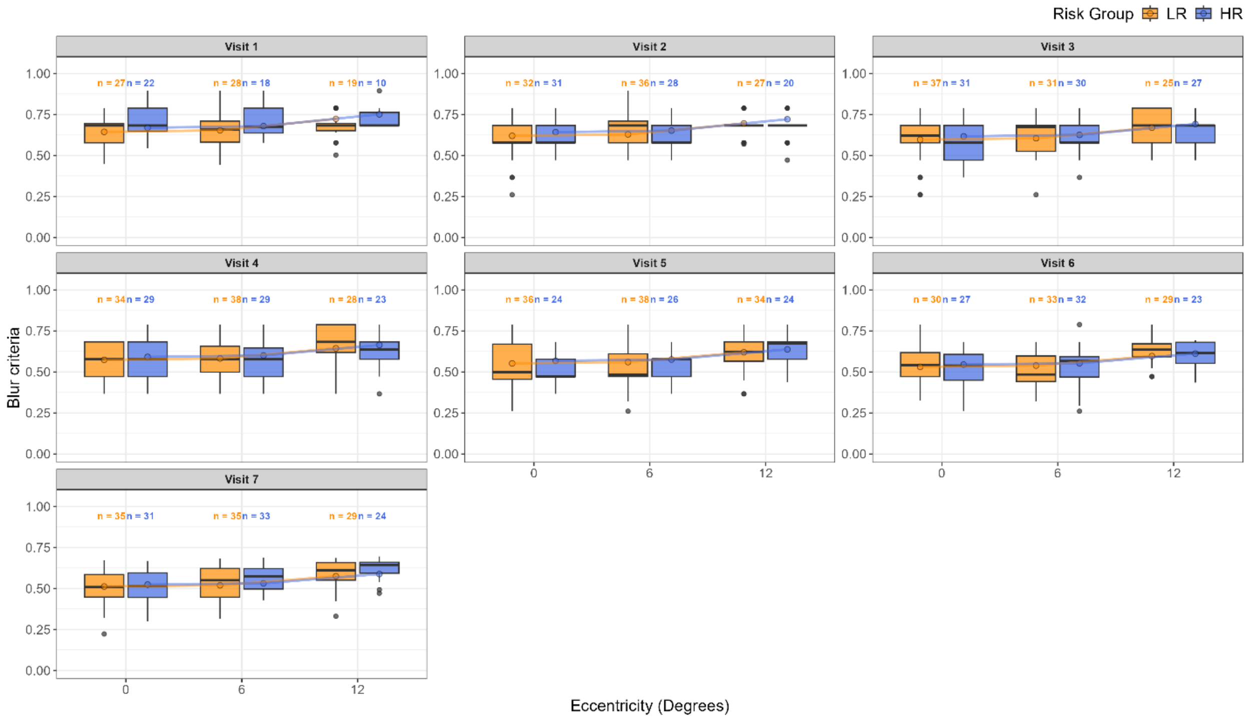

Supplementary Figure S6: Boxplots showing blur discrimination criterion for defocus LR (orange boxes) and HR (blue boxes) groups for defocus blur.

Supplemental Figure S7 (a) shows Q-Q plot, and (b) shows the scatter plots of the Pearson residuals of the models. The Q-Q plot shows a normal distribution of the residuals. However, the model failed to converge. The residuals show a periodic pattern and a deviation of the correlation line (blue) from the reference line(red). See Supplemental File S2 for more details. Nevertheless, the model estimates were close to the raw values, as shown by the linear fits to the estimates in Supplemental Figure S4.   Marginal R^2 was 0.384, and conditional R^2 was 0.58.

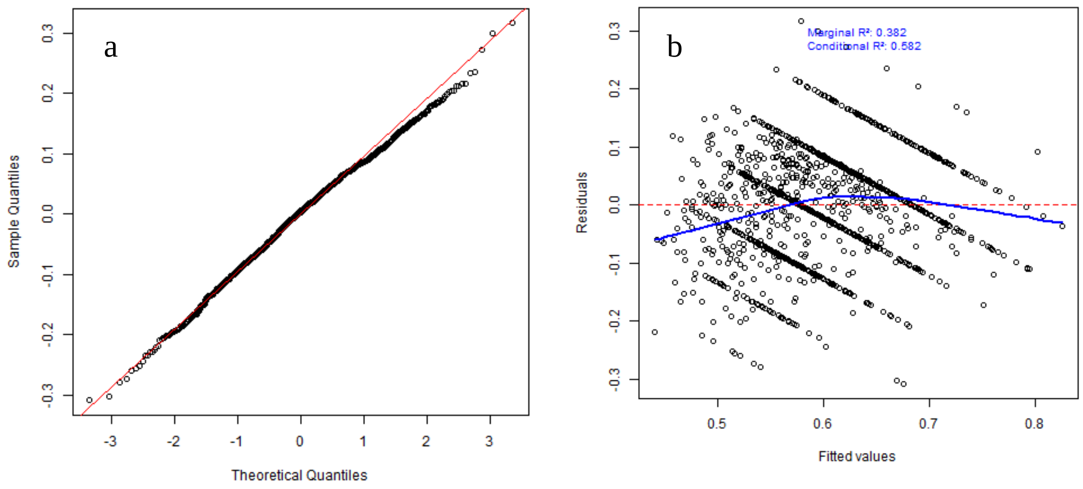

Supplementary Figure S7: Q-Q plot (a) and scatter plot (b) of the Pearson residuals of GLMM fitted blur discrimination criteria for defocus.
