# Supplementary material for: Peripheral Blur Perception in Young Children at Low Risk or High Risk of Myopia: Longitudinal Data
Source: Invest Ophthalmol Vis Sci. 2025 May 28;66(5):40. doi: 10.1167/iovs.66.5.40 (PMC12126130; doi:10.1167/iovs.66.5.40)
Supplement: Supplement 5 [file iovs-66-5-40_s005.pdf]

Blur discrimination criterion for SA

The model summary of the GLMM fitted to blur discrimination criteria for SA is shown in Supplemental Table S4. The values are in log units. Significant values are highlighted in bold.

Supplementary Table S4:
Summary of the GLMM fitted to blur discrimination criteria for SA

	**Blur discrimination criterion for SA**		
Predictors	*Estimates*	*CI*	*p*
(Intercept)	-0.53	-1.06 – -0.01	**0.047**
AgeAtBaseline	-0.06	-0.12 – 0.01	0.102
RiskGrp [1]	0.09	-0.05 – 0.23	0.216
Eccentricity [6]	0.02	-0.01 – 0.06	0.236
Eccentricity [12]	0.05	0.01 – 0.08	**0.007**
Visit	-0.10	-0.11 – -0.09	**<0.001**
RiskGrp [1] × Visit	-0.01	-0.03 – 0.01	0.199
**Random Effects**			
σ^2	0.01		
$\tau_{00\ SubjectID}$	0.02		
ICC	0.70		
$N_{SubjectID}$	98		
Observations	1580		
Marginal R^2 / Conditional R^2	0.639 / 0.893		

There was a significant increase in blur discrimination criterion for SA at 12° eccentricity (0.05 log units, $p = 0.007$) but not at 6° eccentricity (0.02 log units, $p=0.24$) compared to the fovea. Blur discrimination criterion for SA at 6° and 12° eccentricity did not show a significant difference (-0.03 log units, $p=0.39$) on pairwise comparison. There was a significant decrease in blur discrimination criteria with subsequent visits (-0.10 log units, $p<0.001$). There were no other significant associations.

Supplemental Figure S8 shows the boxplots, orange for the LR and blue for the HR group, plotted across eccentricities (degrees). The figure follows the same color scheme as Supplemental Figure S2.

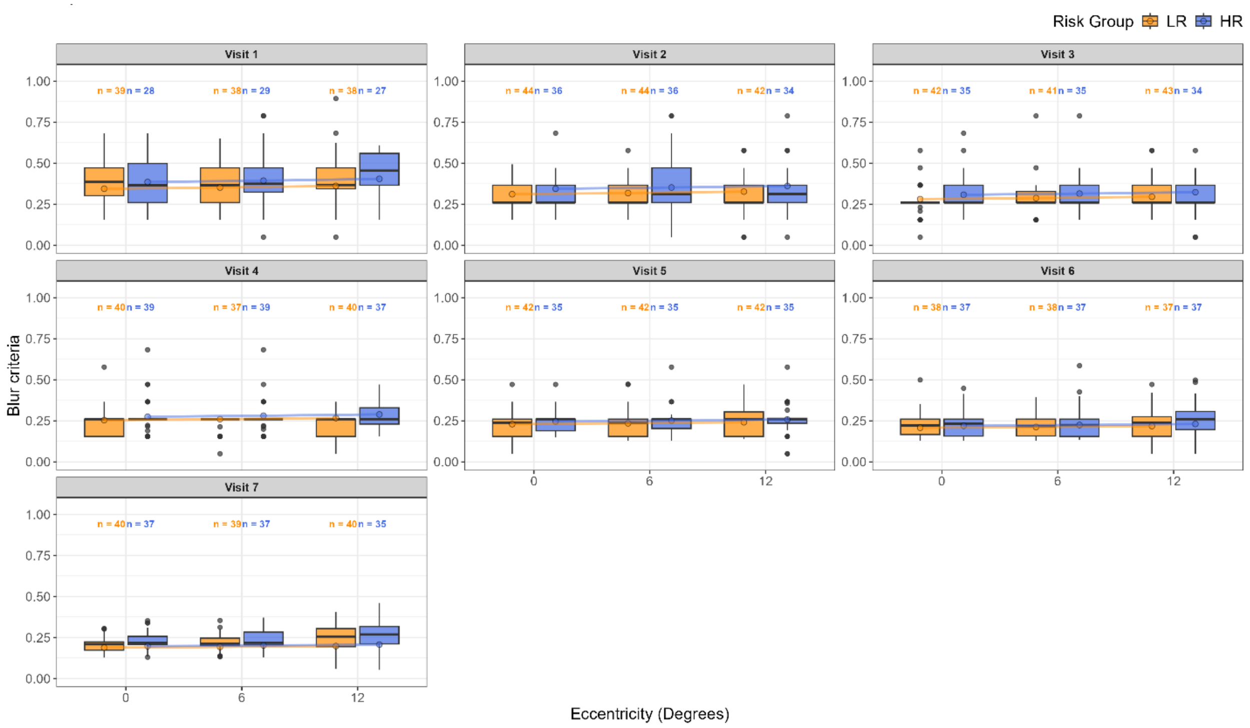

Supplementary Figure S8: Boxplots showing blur discrimination criterion for LR (orange boxes) and HR (blue boxes) groups for SA blur. The lines represent the estimates from the GLMM. The blur discrimination criterion was higher for the peripheral targets compared to the fovea.

Supplemental Figure S9 (a) shows Q-Q plot and (b) shows the scatter plots of the Pearson residuals of the models. The points (black circles) on the Q-Q plot deviate significantly from the diagonal reference line (shown in red). The residuals show a periodic pattern and a deviation of the correlation line (blue) from the reference line(red). See Supplemental File S1 for more details. The estimates generated by the model matched the raw values closely as shown by the linear fits to the estimates in Supplemental Figure S7. Marginal R^2 of 0.64 and conditional R^2 0.89 indicate strong predictability of the model.

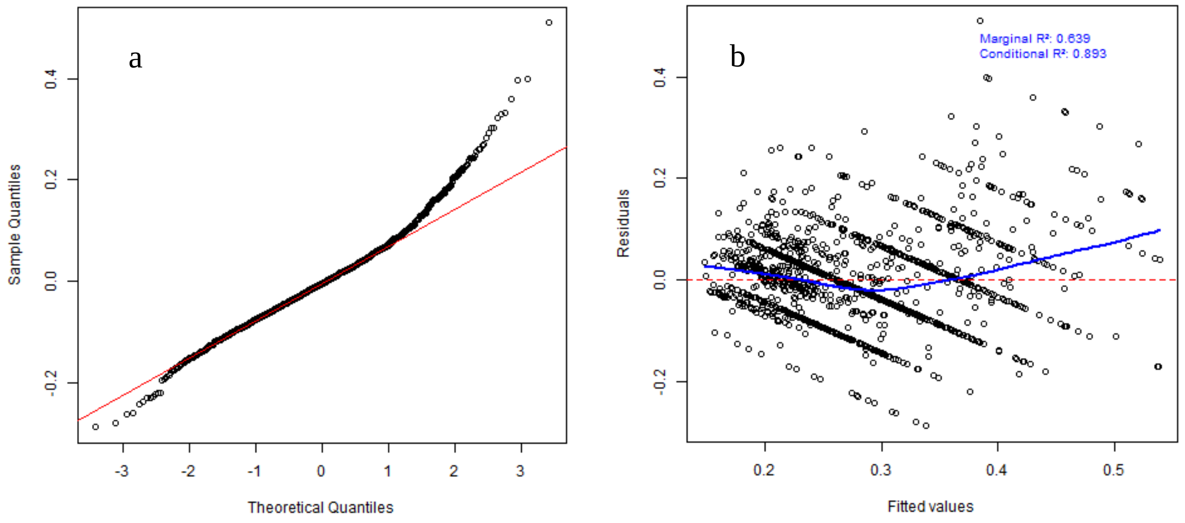

Supplementary Figure S9: Q-Q plot (a) and scatter plot (b) of the Pearson residuals of GLMM fitted blur discrimination criteria for SA.
