# Supplementary material for: Peripheral Blur Perception in Young Children at Low Risk or High Risk of Myopia: Longitudinal Data
Source: Invest Ophthalmol Vis Sci. 2025 May 28;66(5):40. doi: 10.1167/iovs.66.5.40 (PMC12126130; doi:10.1167/iovs.66.5.40)
Supplement: Supplement 6 [file iovs-66-5-40_s006.pdf]

Correlation between axial length and peripheral blur perception

Supplementary Figure S10 illustrates scatter plots showing intrinsic blur for defocus across axial length / Mean anterior Corneal radius (AXL/CR) for each visit in a separate panel. Line of best fit and Pearson correlation r and p are color-coded for eccentricity (blue for 0°, orange for 6° and 12°). The shaded area represents the standard error of the fit. There was weak but significant negative correlation between intrinsic blur and AXL/CR for blur extending beyond 12° at visit 3 (r = -0.343, P= 0.015) and weak but significant positive correlation at visit 6 (r = 0.287, p=0.041).

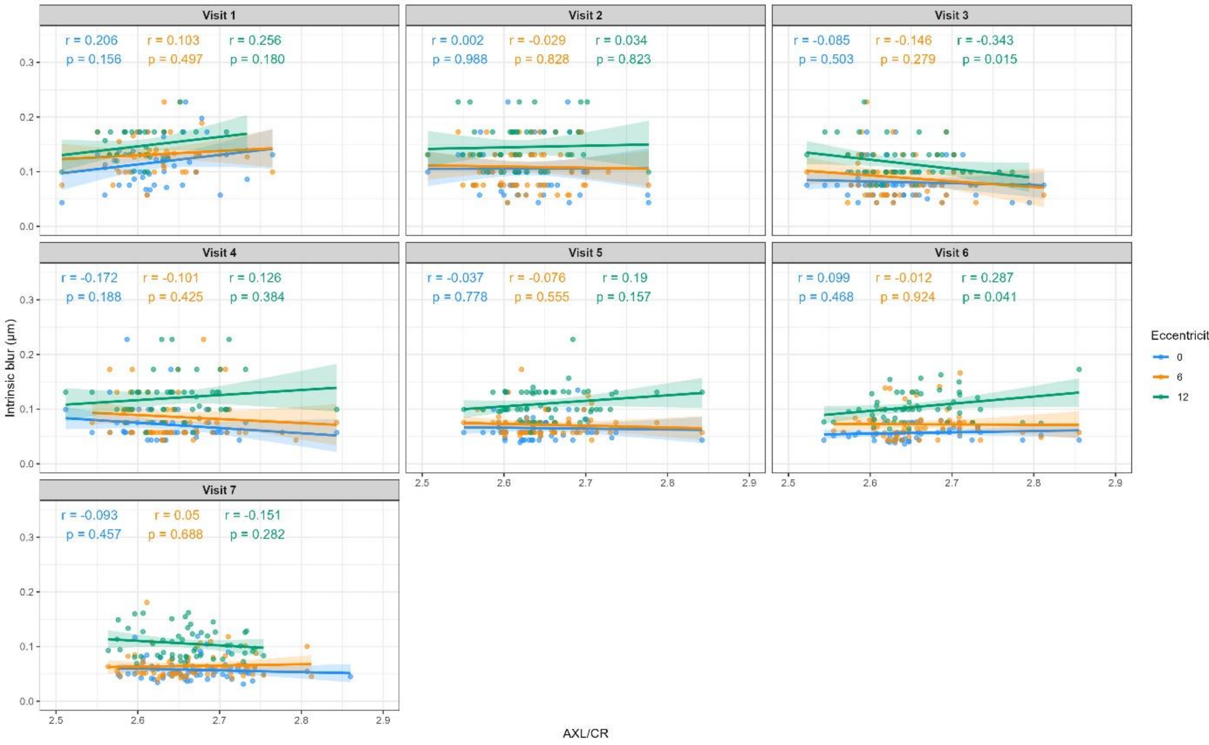

Supplementary Figure S10: Scatter plots showing intrinsic blur for defocus across AXL/CR for each visit.

Supplementary Figure S11 illustrates scatter plots showing intrinsic blur for SA across AXL/CR for each visit in a separate panel, following the same color scheme as Supplementary Figure S10. There was no significant correlation between intrinsic blur for SA and AXL/CR for any eccentricity at any visit (all p>0.05) except blur extended upto the fovea for visit 4 (r =0.308, P = 0.007) and visit 7 (r =0.232, P = 0.046).

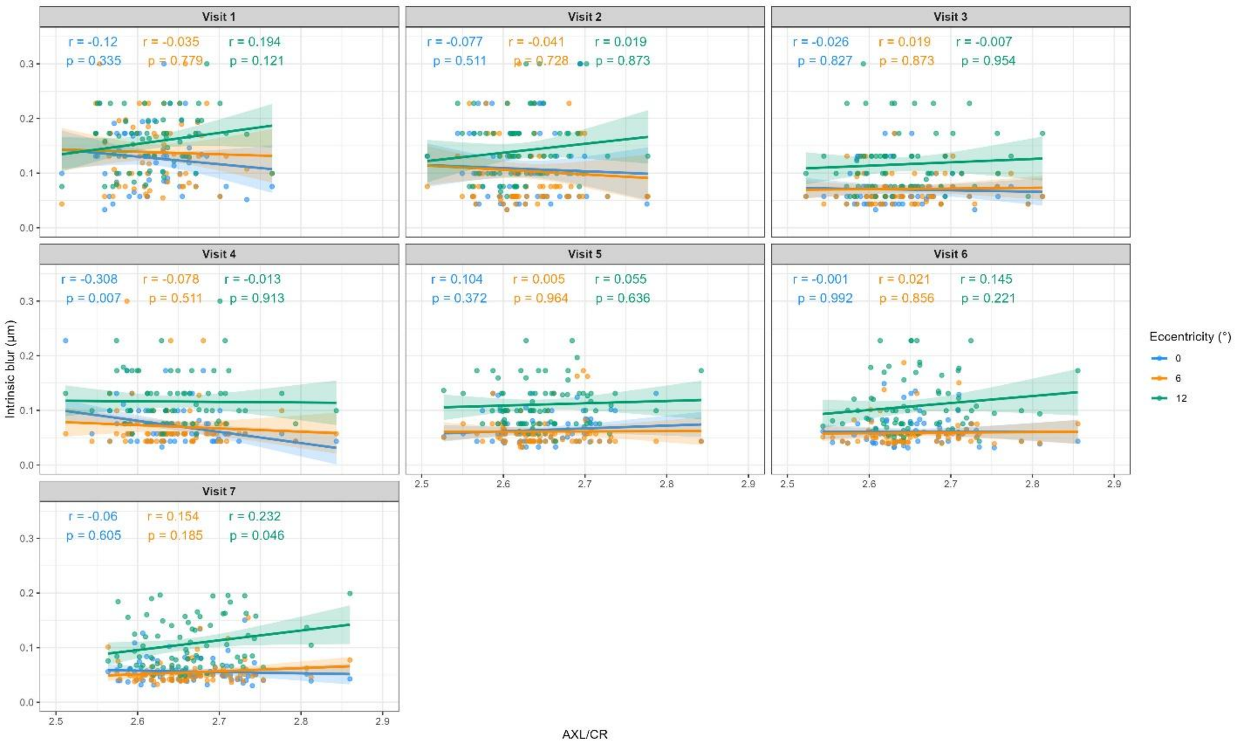

Supplementary Figure S11: Scatter plots showing intrinsic blur for SA across AXL/CR.

Supplementary Figure S12 illustrates scatter plots showing blur discrimination criterion for defocus across AXL/CR for each visit in a separate panel. The correlation between AXL/CR and blur criterion for defocus was not statistically significant (all p>0.05) for visit numbers 1,3,4,5, and 6. There was a weak but statistically significant positive correlation between AXL/CR and blur criterion for defocus for eccentricity beyond 12° for visit 2 (r = 0.382, p = 0.009) and visit 5 (r = 0.391, p=0.003).

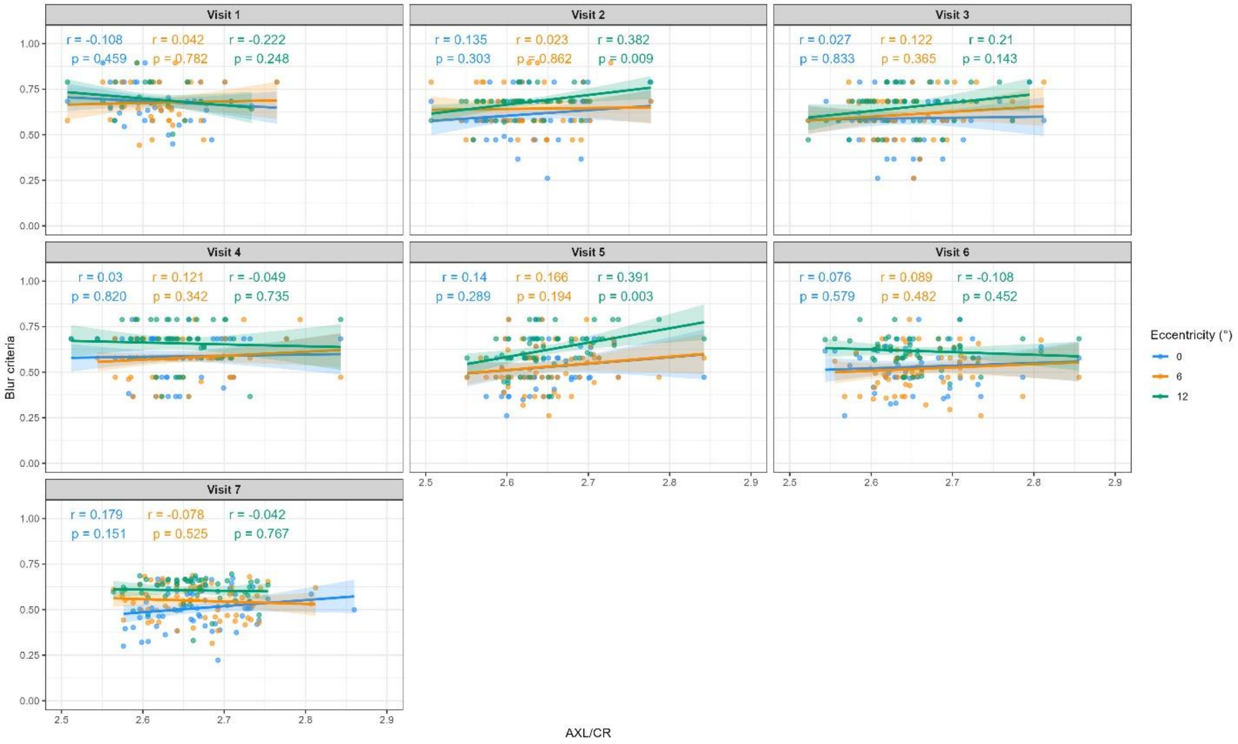

Supplementary Figure S12: Scatter plots showing blur discrimination criterion for defocus across AXL/CR for each visit.

Supplementary Figure S13 illustrates scatter plots showing blur discrimination criterion for SA across AXL/CR for each visit in a separate panel. The correlation between axial length and blur criterion for SA was not statistically significant (all p>0.05) for visit numbers 1,2, and 4 to 7 . There was a weak but statistically significant positive correlation between AXL/CR and blur criteria for SA for eccentricity beyond 12° (r = 0.254, p = 0.030) at visit 3.

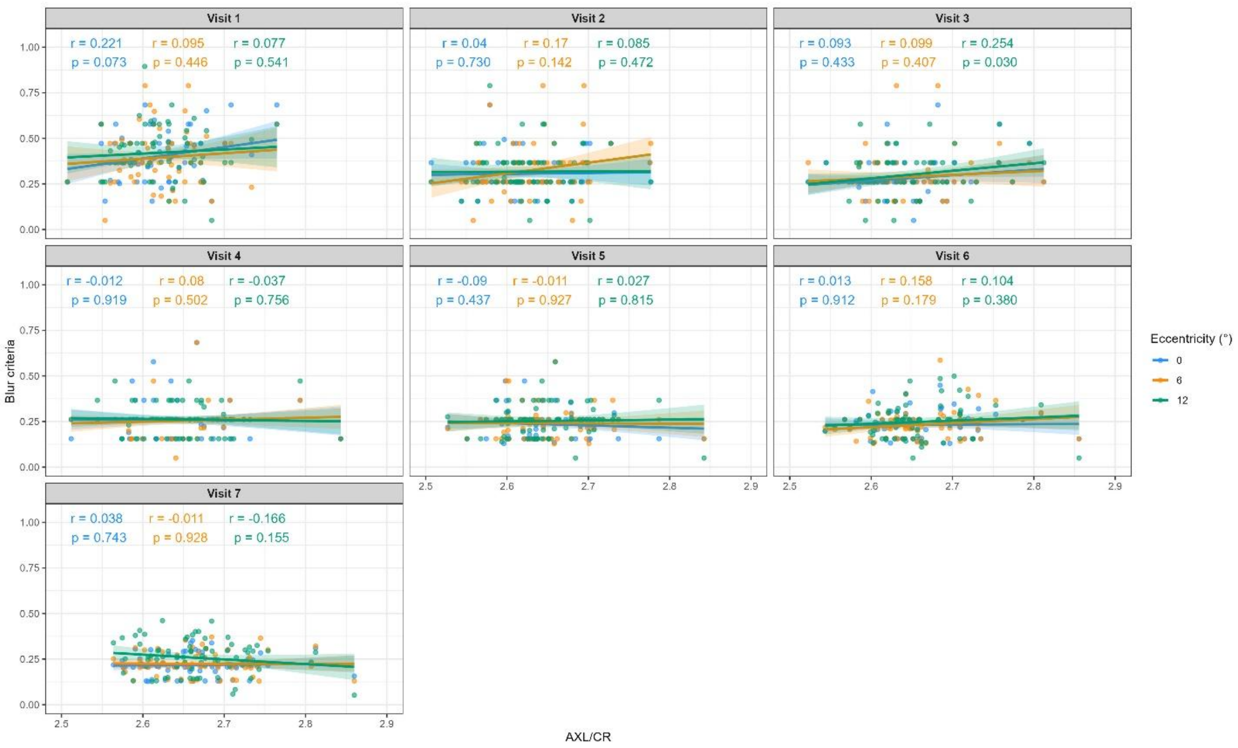

Supplemental Figure S13: Scatter plots showing blur discrimination criterion for SA across AXL/CR.

Overall peripheral blur perception was not correlated with the AXL/CR in both HR and LR groups of children.
