# Supplementary material for: Peripheral Blur Perception in Young Children at Low Risk or High Risk of Myopia: Longitudinal Data
Source: Invest Ophthalmol Vis Sci. 2025 May 28;66(5):40. doi: 10.1167/iovs.66.5.40 (PMC12126130; doi:10.1167/iovs.66.5.40)
Supplement: Supplement 7 [file iovs-66-5-40_s007.pdf]

Correlation between refractive error and peripheral blur perception

Supplementary Figure S14 illustrates scatter plots showing intrinsic blur for defocus across cycloplegic refractive error, M (D) for each visit in a separate panel. Line of best fit and Pearson correlation r and p are color-coded for eccentricity (blue for 0°, orange for 6° and 12°). The shaded area represents the standard error of the fit. There was only a marginal significance in correlation between intrinsic blur for defocus and M at visit 6 for eccentricity beyond 12° (r = -0.276, p=0.047).

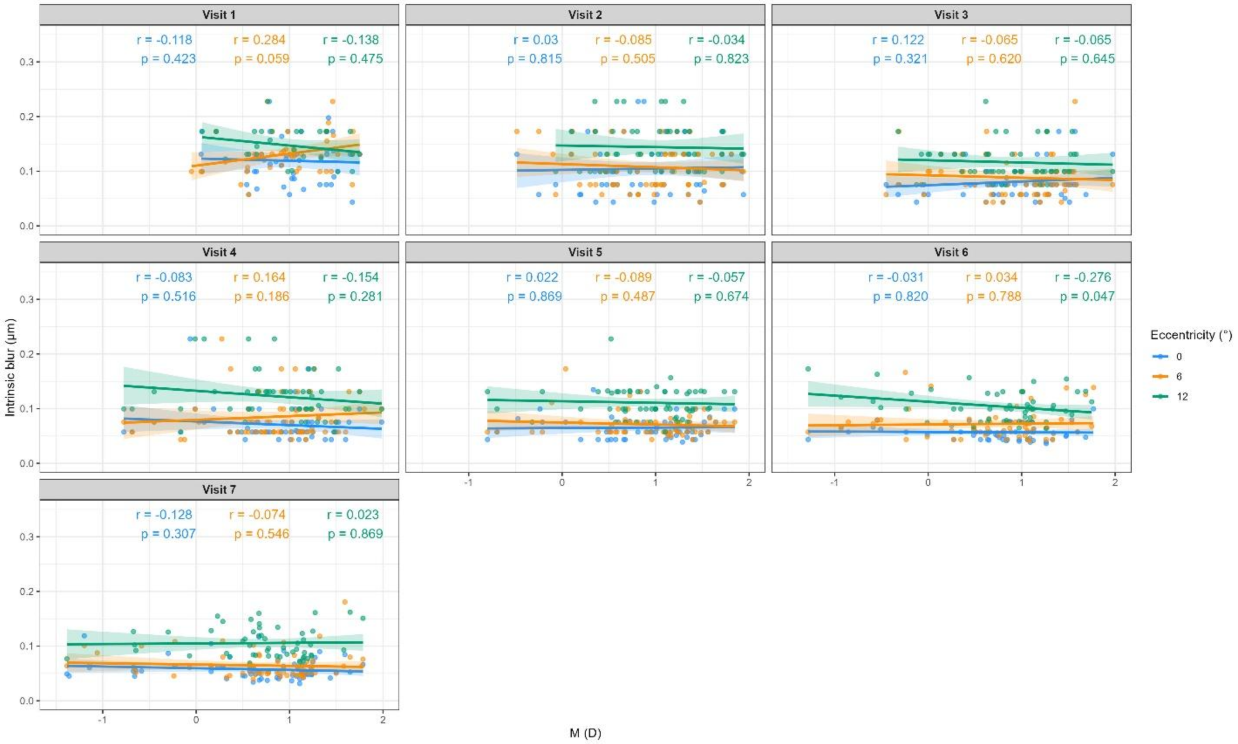

Supplementary Figure S14: Scatter plots showing intrinsic blur for defocus across refractive error M for each visit.

Supplementary Figure S15 illustrates scatter plots showing intrinsic blur for SA across M for each visit in a separate panel following same color scheme as Supplementary Figure S10. There was no significant correlation between intrinsic blur for SA and M for any eccentricity at any visit (all p>0.05).

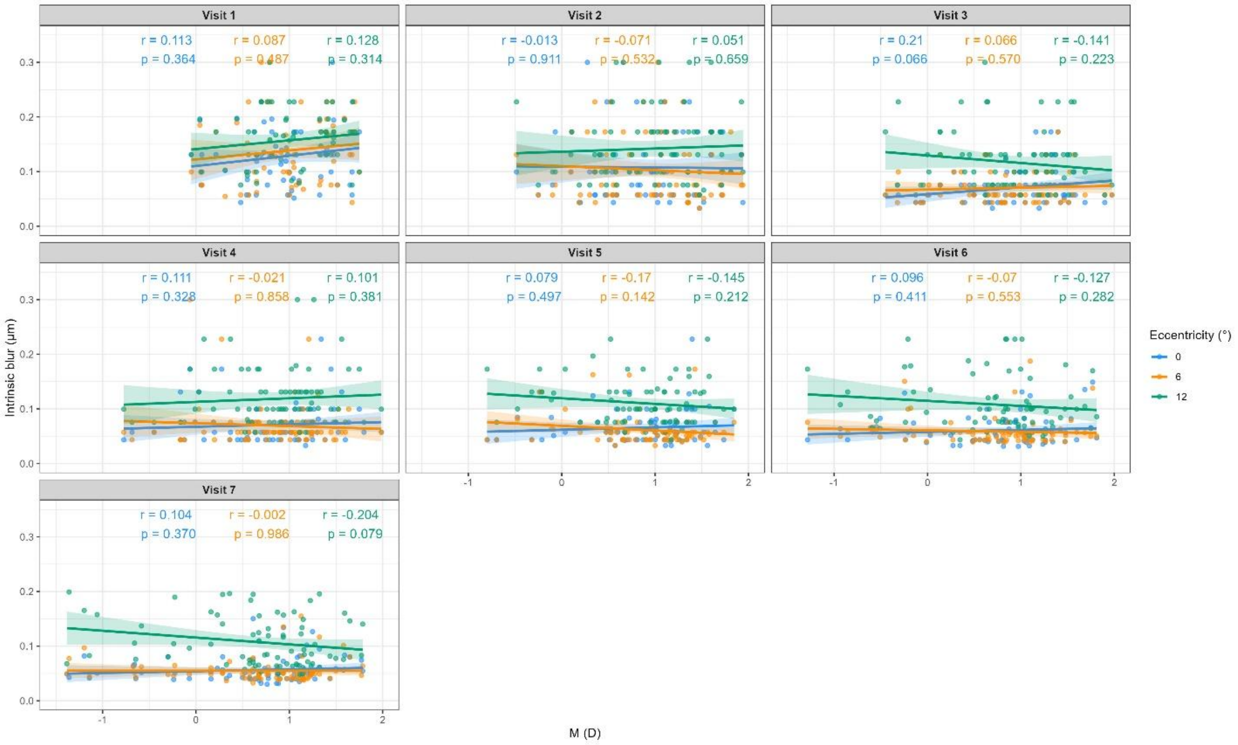

Supplementary Figure S15: Scatter plots showing intrinsic blur for SA across refractive error, M.

Supplementary Figure S16 illustrates scatter plots showing blur discrimination criterion for defocus across M for each visit in a separate panel. The correlation between M and blur criterion for defocus was not statistically significant (all p>0.05) for any eccentricity at any visit.

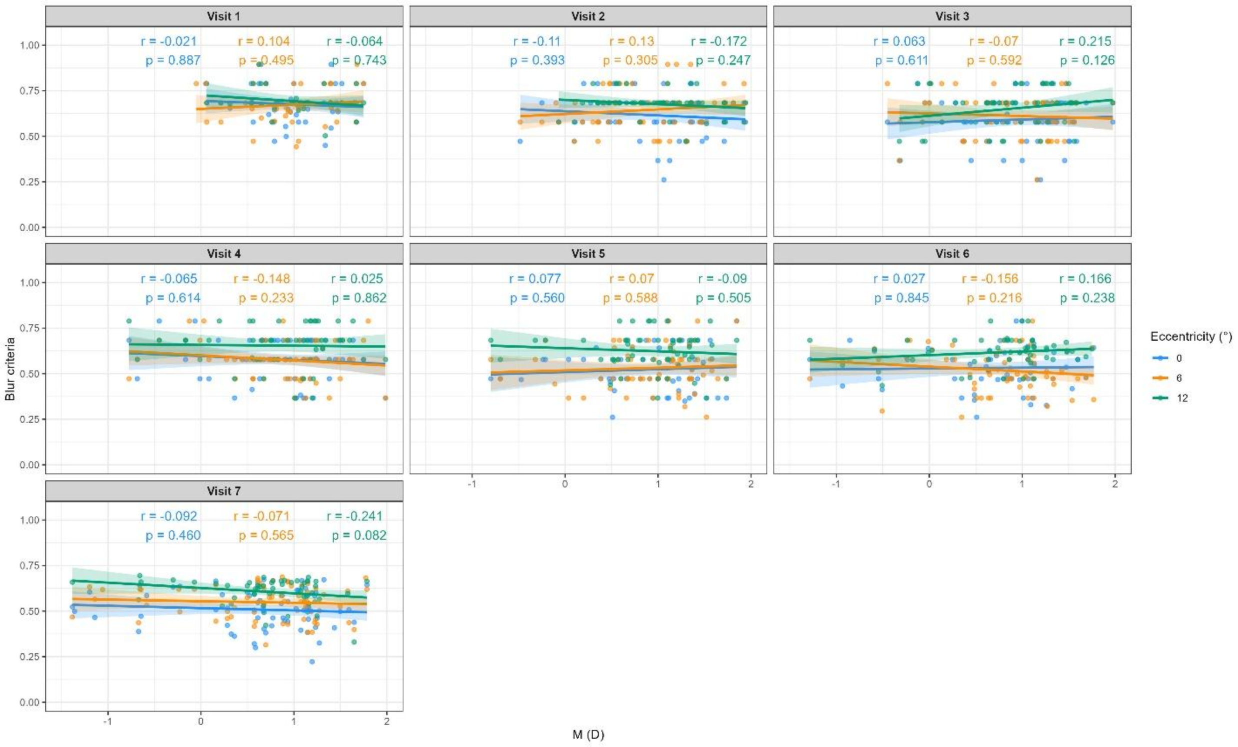

Supplementary Figure S16: Scatter plots showing blur discrimination criterion for defocus across AXL/CR for each visit.

Supplementary Figure S17 illustrates scatter plots showing blur discrimination criterion for SA across M for each visit in a separate panel. The correlation between axial length and blur criterion for SA was not statistically significant (all p>0.05) for visit numbers 1 to 6. There was a weak but statistically significant positive correlation between M and blur criteria for SA for blur extending up to the fovea (r = 0.26, p = 0.039) at visit 7.

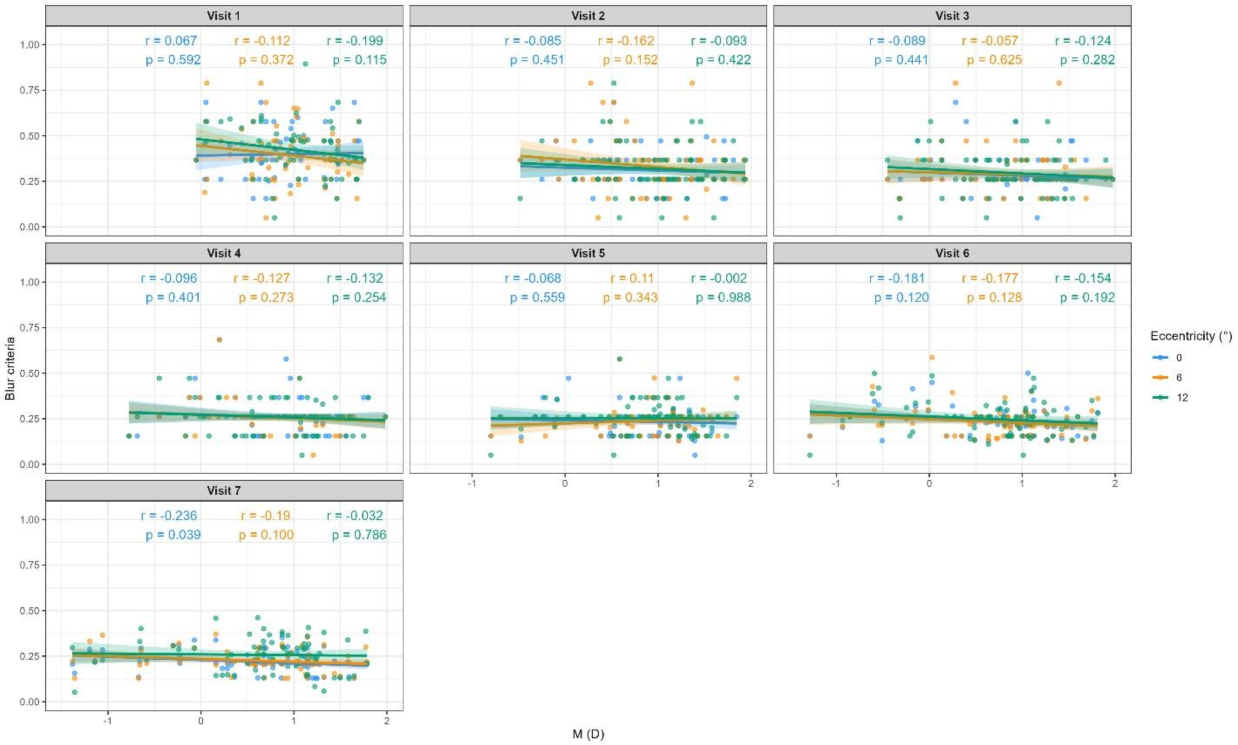

Supplemental Figure S17: Scatter plots showing blur discrimination criterion for SA across refractive error M.

Overall peripheral blur perception was not correlated with the M in both HR and LR groups of children.
